# Friedreich's ataxia patient pathway in Europe

**DOI:** 10.3389/frhs.2026.1817584

**Published:** 2026-05-28

**Authors:** Julie Vallortigara, Julie Greenfield, Barry Hunt, Bart-Jan Schuman, Deborah Hoffman, Suzanne Booth, Carola Reinhard, Holm Graessner, Antonio Federico, Vinciane Quoidbach, Steve Morris, Paola Giunti

**Affiliations:** 1 Ataxia Centre, Department of Clinical and Movement Neurosciences, UCL Queen Square Institute of Neurology, London, United Kingdom; 2 Ataxia UK, London, United Kingdom; 3 Friedreich Ataxie Förderverein e.V., Pliening, Germany; 4 Takeda Pharmaceuticals, Cambridge, MA, United States; 5 Centre for Rare Diseases and Institute of Medical Genetics and Applied Genomics, University Hospital Tübingen, Tübingen, Germany; 6 Department of Medicine, Surgery and Neurosciences, Medical School, University of Siena, Italy; 7 European Brain Council, Brussels, Belgium; 8 Primary Care Unit, Department of Public Health and Primary Care, University of Cambridge, Cambridge, United Kingdom

**Keywords:** care pathway, costs, friedreich's ataxia, patient survey, specialist centre, health service use, rare disease

## Abstract

**Background:**

Friedreich's ataxia (FA) is a rare progressive and multi systemic neurodegenerative disorder characterized by loss of coordination, typically resulting in loss of ambulation. Cardiomyopathy, diabetes mellitus, and scoliosis are common and serious manifestations of the disease. This project explores the FA patient pathways, healthcare use and costs, and compares people's care experiences attending specialist ataxia centres (SAC) with care in non–specialist settings in three European countries.

**Methods:**

We collected data in the UK, Germany and Italy using a patient survey, to gather information about the diagnosis and the management of the ataxias in SAC and non-SAC settings, healthcare visits, and patient's satisfaction. We compared per-patient mean resource use for each contact type and health service cost, stratifying patients by SAC attendance.

**Results:**

Our cohort is 97 patients with FA over 16 years old, living in UK (*N* = 27), Germany (*N* = 14) and Italy (*N* = 56) with a confirmed diagnosis of FA. This cohort valued the SAC services in understanding their condition, offering opportunities to take part in research, and delivering care adapted to their needs. Our results highlight the patient's appreciation for SAC services compared to non-SAC without any significant change in costs. We identified similar barriers for patients in accessing SACs in the selected countries.

**Conclusions:**

This study shows the value of SAC specifically for the management of complex rare conditions like FA. The outcomes of our work will be used for policy recommendations on how to improve treatment and care for people with rare neurological disorders across Europe.

## Introduction

Friedreich's ataxia (FA) is a rare progressive and systemic neuromuscular disease characterized by loss of coordination, typically resulting in loss of ambulation (1). Cardiomyopathy, diabetes mellitus, scoliosis and pes cavus are common extra neurological and serious manifestations of the disease. Speech and swallowing are affected, hearing impairment is also common, while loss of vision is less frequent. FA affects ∼1:20,000 lives births in western Europe, where it is the most prevalent form of inherited ataxia (2). Clinical course and rate of progression are variable and are influenced by age at disease onset (3). FA typically presents between 5 and 15 years of age; these patients have a shortened life expectancy, with the most common cause of death being cardiac failure. Late onset patients (>25 years of age at disease onset) are considered atypical and exhibit a disease course that includes slower progression, milder manifestations of ataxia and dysarthria, and few non-neurological symptoms.

Up to now, there were no disease modifying treatments specifically for FA available to patients in Europe. However, a first disease-modifying treatment (DMT), Omaveloxolone, a Nrf 2 activator, was finally approved by the FDA (Food and Drug Administration) (in 2023), the EMA (European Medicines Agency) (in 2024), the MHRA (Medicines and Healthcare products Regulatory Agency) (in 2025) and other regulators globally (approved in Canada) for people aged 16 and above with FA (4–7). This development has the potential to slow disease progression and fundamentally alters long-term care trajectories. Although the drug is available in countries such as the USA, in many European countries, it is undergoing health technology assessment (HTA) processes and pricing and reimbursement negotiations to determine its clinical and economic value within national healthcare systems. As a result, this treatment is currently either not yet accessible through standard care pathways or is available to a limited subset of patients under national early access or compassionate use programmes.

At the time of this study, the management of FA was largely focused on the treatment of symptoms and comorbidities. Because of the serious consequences of the disease, patients should be diagnosed as early as possible. An early diagnosis can help healthcare professionals (HCPs) manage the symptoms of FA and allow for the building of a multidisciplinary care team (MDT) tailored to a patient's individual needs (8). Multidisciplinary care is essential to mitigate the impact of this progressive disease and support patients in maintaining function and quality of life. For example, regular monitoring of cardiac complications and early diagnosis of diabetes - both of which can be managed with established therapies – are critical components of care (5). For all progressive ataxias, including FA, the early institution of physiotherapy plays a crucial role in preventing spasticity, spasms, joint stiffness, and contractures (8). Complementary interventions from occupational therapists (OT) help preserve independence, while speech and language therapists (SLT) are essential for maintaining communication and safe swallowing, thereby reducing risks related to and aspiration (8, 9). In addition, many patients benefit from timely intervention from other specialists, including cardiologists (for heart complications), urologists (for bladder issues), gastroenterologists (for bowel complications), otologists (for vertigo and hearing loss), and ophthalmologists (for visual impairments).

The new treatment paradigm does not diminish the need for multidisciplinary care; rather, it reinforces the role of specialist centres as anchors of expertise in disease monitoring, treatment initiation and coordination, and long-term management. As care models evolve, ongoing monitoring of the patient care pathway will be essential to assess therapeutic impact, ensure timely access to services, and adapt multidisciplinary support accordingly. To better understand how care is currently delivered, we evaluated the patient pathways in Specialist Ataxia Centres (SACs) in three countries which reflect the different nature of the services in the selected countries. However, they share a common background in seeing patients with rare diseases and the efforts to translate research impact into the service provided within their own specialised service. The first centre established with research and translation management was the SAC in the UK in 2005 (10, 11). Italy, along with other European countries, has since adopted similar care models for the diagnosis and management of ataxias.

Improving care for people living with FA required capturing their lived experiences – including the burden of disease on everyday life, the structure of their care pathways, and their expectations for future treatment options. While several qualitative studies have documented challenges that FA patients experience related to diagnosis and disease management, (12, 13) there is a lack of comparative research examining patient experiences across different healthcare systems. The patient voice – particularly their interactions with healthcare professionals and ability to access more specialised, adapted services from primary care – provides essential insights into unmet needs and future priorities. Understanding how patients navigate the care system, and where challenges remain, is critical to building responsive and effective services.

In this study, we analysed data collected from patients living with FA in the UK, Germany, and Italy using a structured questionnaire. Some data from this survey related to all kind of ataxias has recently been published (14, 15). The analysis done on the three countries highlighted differences in the patient care pathway with referral to SACs throughout primary care services and local neurologists in the UK whereas in Germany and Italy we noted various ways for patients to access specialised services including research studies and self-referrals. We discussed the different roles SACs can play in terms of management of the symptoms vs. clinical research activities, with SACs in Italy and Germany focusing more on diagnosis and clinical trials and less on the management compared to the UK. As FA is the most common and complex inherited ataxia, we have carried out an in-depth analysis that includes new unpublished data. We explored patient care pathways, access to treatment, experiences within SACs, cost associated with resource use, and views on current gaps in care. Our findings also shed light on the role of SACs in supporting multidisciplinary interventions and preparing for the integration of DMT into routine clinical practice.

## Material and methods

We focused our study on three countries: UK (two centres: London and Sheffield), Germany (nine centres based in University Hospitals: Luebeck, Munich, Tuebingen, Bonn, Essen, Aachen, Berlin, Düsseldorf, Magdeburg), and Italy (eleven centres including 6 in University Hospitals: Turin, Florence, Siena, Naples, Messina, Rome-Sapienza and the remaining in IRCCSs (Scientific Institute for Research, Hospitalization and Healthcare, in Italian: Istituti di Ricovero e Cura a Carattere Scientifico): Milan, Pisa, Genova, Bologna, Rome-Bambin Gesu).

## Patients

FA patients (or carers of patients, as proxy respondents), who were 16 years old or over participated in the survey. In each country patients’ groups or organisations helped with the recruitment of participants. The survey in the UK was disseminated online through the patient group Ataxia UK channels (mailing list, website, magazine and on social media). In Germany and Italy, it was disseminated via both patients’ groups channels and clinicians working at the SACs. For Germany, the patients’ groups were DHAG (Deutsche Heredo Ataxie Gesellschaft), Friedreich Ataxie Förderverein e.V., and for Italy the patients’ group was AISA (Associazione Italiana per la lotta alle Sindromi Atassiche).

## Survey design

A working group with patient group representatives, a specialist ataxia neurologist, a specialist ataxia nurse, a health economist and representatives of pharmaceutical companies was involved in the design of this survey. The final version of the survey had 64 non-obligatory questions relating to the following topics: demographics, diagnosis, referrals, patients’ encounters with healthcare professionals, treatments received and patient satisfaction. The context of the study and the medical terms used in the survey were explained at the beginning of the survey (See Appendix 1). The survey was first rolled out in the UK, used as a pilot. Minor modifications were made following feedback from patients’ representatives and clinicians during focus groups in Germany and Italy, including the addition of questions on mobility status and referral details to SACs and MDT clinics, and clarification of wording for cross cultural relevance as well as changing the lay out for some questions. The revised survey was then translated and disseminated in these two countries (Appendices 2, 3).

## Data collection

The survey was put online on the SurveyMonkey platform and rolled out during the following periods: March–May 2019 in the UK, February–October 2020 in Germany, and May–September 2021 in Italy. The responses were then exported from SurveyMonkey and collated in Microsoft Excel for data cleaning and analysis. The data cleaning process involved the removal of all respondents who did not provide informed consent or positive responses to all three screening questions for an anonymised version of the database (questions 3,4,5; see Additional file 10: Appendix 1). Where the respondent gave contradictory responses in different questions, the responses to those questions were removed from the analysis. Incomplete surveys, where some questions were left unanswered, were not removed from the analysis. The number of respondents was reported for each individual question.

## Ethics and consent

Ethical approval was obtained for this study in the UK (REC; reference 19/EE/0030). For the two other countries, as the survey was anonymised no ethical approval was needed. Consent was obtained online, with the act of submitting the survey considered as consent to take part in the study.

## Data analysis

Responses to survey questions were stratified by attendance at SAC. People who had never been to a SAC were grouped in the ‘non-SAC’ group; people who are currently going to a centre were grouped in ’SAC’ group. There was a third group of people who used to go to a SAC but no longer go; they were grouped in the ‘Used to SAC’ group. This group was not included in the statistical analysis because they experienced a mixed care pathway. However, the results of the survey for this group have been used to identify barriers to continue to access SACs. In questions with responses measured on a Likert scale, the affirmative responses were grouped using the responses `very positive/positive/slightly positive’ or `very effective/effective/slightly effective’ or ’strongly agree/agree/slightly agree’ and negative responses were considered to be either ‘very negative/negative/slightly negative’ or ‘very ineffective/ineffective/slightly ineffective’ or ’strongly disagree/disagree/slightly disagree’, respectively. For questions towards the end of the survey about overall symptoms management and care delivered, affirmative responses were considered to be ‘best it could be/very well/quite well/adequately’. Results were prepared as tabulated descriptive statistics and presented as numbers (n) and percentage (%) of total respondents per question. The questions were treated independently, and statistical tests were performed to compare results between the ‘non-SAC’ and ’SAC’ groups for each question using the Chi-Square Pearson test.

## Resource use and costs

Participants were asked to record the number of health care contacts they had received in the preceding 6 (in the case of the UK) or 12 (in Germany and Italy) months specifically to manage their ataxia. Participants were asked to record the number of SAC visits, and the number of contacts with the family doctor, hospital outpatient clinic visits with a neurologist (not at a SAC), hospital inpatient stays, emergency room visits, physiotherapy appointments, speech and language therapy appointments, occupational therapy appointments, and other consultant specialist visits (e.g., an ophthalmologist, an ear, nose and throat specialist, urologist, gastroenterologist and others). Figures for the UK were multiplied by two to give 12-month estimates, commensurate with the other two counties (16). Unit costs for each type of contact were obtained from local providers or published sources (17, 18). All costs were calculated in 2023; unit costs for the UK were reported in UK£ and converted into € using GDP Purchasing Power Parities; unit costs for Germany and Italy were reported in €; prices were converted into 2023 values using published inflation indices.

We compared average resource use for each contact type, and costs, over a 12-month period, stratifying patients by whether they were currently attending a SAC or had never attended a SAC. We tested for significant differences in mean resource use and costs between the SAC/non-SAC groups in each country using unadjusted and adjusted ordinary least squares regression analysis, the latter controlling for age, sex, number of symptoms experienced as a result of ataxia, and whether or not the patient had comorbidities. We used responses to question 43 in the survey (number of symptoms experienced as a result of FA) as proxy to inform the degree of severity of the disease. We also tested for significant differences in mean contacts and costs between countries separately for Non-SAC and SAC groups using adjusted ordinary least squares regression analysis, controlling for age, sex, number of symptoms experienced as a result of ataxia, and whether or not the patient had comorbidities.

## Patient travel

As part of the surveys, we also asked respondents who reported using the SAC to record the main mode of transport they used to travel to the centre and travel time from home to the centre. We also asked all respondents how long it took to travel one-way to receive ataxia care by a neurologist based in general neurology clinic (not at a SAC), and for Italy and Germany the main mode of transport they used. We present these data separately according to whether patients reported they were currently attending a SAC or had never attended a SAC.

## Results

### Demographics

The characteristics of the cohort of respondents are summarised in Table 1. Most surveys were filled by the people diagnosed with FA rather than carers. The proportion of participants who were currently attending a SAC or not was different between the three countries with higher proportion of respondents in non-SAC group in the UK and higher proportion in SAC group in Germany and Italy. There were no respondents in non-SAC group in Germany. All respondents were affected by their ataxia at the time of the survey with 64.3% and 75% using a wheelchair permanently in Germany and Italy respectively, followed by 21.4% and 10.7% using walking aids in same both countries (Supplementary Tables 1a-b; data not available for the UK).

**Table 1:** Survey respondent demographics.

Characteristic	Responses N (%)	UK	Germany	Italy
**Respondent type**	*Patient*	20 (74.1)	11 (78.6)	45 (80.4)
	*On behalf of the patient*	7 (25.9)	3 (21.4)	11 (19.6)
	*Total*	27 (100)	14 (100)	56 (100)
**Age distribution**	*16–29*	6 (24)	9 (64.3)	11 (19.6)
	*30–59*	15 (60)	5 (35.7)	43 (76.8)
	*60–80*	4 (16)	0 (0)	2 (3.6)
	*80+*	0 (0)	0 (0)	0 (0)
	*Total*	25 (100)	14 (100)	56 (100)
**Gender**	*Female*	17 (68)	5 (35.7)	36 (64.3)
	*Male*	8 (32)	9 (64.3)	20 (35.7)
	*Total*	25 (100)	14 (100)	56 (100)
**Attendance to SAC**	*Never been*	12 (66.7)	0 (0)	5 (11.1)
	*Currently going*	3 (16.65)	12 (85.7)	30 (66.7)
	*Used to go*	3 (16.65)	2 (14.3)	10 (22.2)
	*Total*	18 (100)	14 (100)	45 (100)

### Living situation

In the UK, 10 (42%) participants lived alone and 14 (58%) lived with a spouse or family members, similarly in Germany a majority of respondents lived with a spouse or family members (*n* = 7, 50%), or lived alone (*n* = 4, 28.6%). Participants from Italy lived for a majority with a carer who was a friend, partner or family member (non-professional, *n* = 38, 72%).

### Working situation

In the UK 45.5% participants reported an early retirement because of their ataxia and 27.3% never worked (Supplementary Table 2A). This makes the proportion of people in the UK who are not working higher (72.8%) compared with Germany (5.3%) and Italy (28.8%). In Germany and Italy, 26.3% and 16.7% were still working, and 26.3% and 12.1% reduced their working hours because of their ataxia respectively (Supplementary Tables 2B-C). Only 5% in Germany and 14% in Italy received welfare support (data not available for the UK). In the UK the SAC group was small however it's interesting to see that one participant still worked with work arrangement whereas no one worked in non-SAC or Used to SAC group. In Italy, we note the same proportion of participants was still at work in SAC group (16.7%) and non-SAC group (16.7%); the group Used to SAC show a higher proportion of 23.1%.

### Reaching a diagnosis

A majority of FA patients in the UK, Germany and Italy received their diagnosis more than 5 years (Supplementary Figures 1a-c). At that time of first diagnosis, most respondents were already affected by their ataxia with ‘occasional difficulties’ for their daily activities for 58.3%, 69.2% and 54.5% of the respondents in the UK, Germany and Italy respectively, and ‘frequent difficulties’ for (20.8%, 7.7% and 16.4% in the same countries. In Germany and Italy, patients also reported the impact of their ataxia at the time of the survey with 50% and 55.6% having constant problems with their daily activities in Germany and Italy respectively. Overall, 12 out of 13 people (92%) and 36 out of 42 (85.7%) in German and Italy experienced over time (between the time of their first diagnosis and the time of the survey) a progression of impact on daily activities. In Italy, there was no difference in this outcome related to attendance to SAC or not (data not available for Germany and UK).

Most patients received their diagnosis by the neurologist in the UK (*N* = 21; 84%), in Germany (*N* = 11; 78.6) and Italy (*N* = 48; 84.2%). In Germany only 15.4% (*N* = 2) of participants went to see a neurologist in a SAC as first referral, in Italy this proportion was higher with 54.5% (*N* = 24). For Germany and Italy, we also asked where participants got their FA diagnosis confirmed by genetic testing; in Germany the responses were 5 in a SAC (35.7%), 5 in non-specialist service (35.7%), 3 in other services detailed in the caption (Supplementary Table 3a). In Italy, there was more people getting their confirmed diagnosis in a SAC (58.9%, *N* = 33) than in non-specialist services (35.7%, *N* = 20) (Supplementary Table 3b).

The time spent between the first visit to the neurologist to the confirmation of the FA diagnosis by genetic testing was variable for the participants from Germany with 4 waited between 6 months and 1 year (30.8%), 3 up to 6 months (23.1), 2 received the specific diagnosis when they saw the neurologist (15.4%) and 2 had to wait more than 5 years (15.4%). In Italy, participants experienced less waiting time with top responses being when they saw the neurologist (*N* = 16, 29.1%), up to 6 months (*N* = 11, 20%), and between 6 months and one year (*N* = 13; 23.6%) (data not available for the UK).

### Patient pathway

#### See a neurologist

Looking at the time spent between the first symptoms and the first appointment with a neurologist, there was a higher proportion of participants who saw a neurologist within 6 months in Germany and Italy compared to the UK (Table 2).

**Table 2:** Time spent between the first symptoms and first appointment with a neurologist.

Answer choices	Responses UK N (%)	Responses Germany N (%)	Responses Italy N (%)
I was referred to a neurologist immediately	5 (20)	0	0
Up to 6 months	3 (12)	8 (57.2)	27 (60)
Between 6 months and 1 year	4 (16)	2 (14.3)	0
Between 1 and 2 years	5 (20)	2 (14.3)	0
Between 2 and 5 years	3 (12)	1 (7.1)	1 (2.2)
More than 5 years	4 (16)	0	2 (4.4)
Unsure	1 (4)	1 (7.1)	15 (33.4)
Total	25 (100)	14 (100)	45 (100)

#### Access to a SAC

In terms of referral to a SAC, a majority were referred by the local Hospital neurologist in the three countries, followed by the GP and other ways (Table 3). A majority of participants were referred more than 5 years ago (*N* = 4, 44.5% in the UK; *N* = 7, 70% in Germany; *N* = 22, 88% in Italy).

**Table 3:** HCPs who referred people to a SAC.

Answer choices	Responses UK N (%)	Responses Germany N (%)	Responses Italy N (%)
GP	2 (22.2)	4 (28.6)	8 (21.6)
Other primary care HCPs	0 (0)	-	-
Hospital neurologist	5 (55.6)	4 (42.9)	16 (43.3)
Other	2 (22.2)	4 (28.6)	6 (16.2)
Unsure	0 (0)	0	7 (18.9)
Total	9 (100)	14 (100)	37 (100)

Other UK: neurologist at SAC during a research study.

Other Germany: research study, Social-paediatrics centre, self-referral.

Other Italy: physiatrist, self-referral, orthopaedist, psychiatrist.

We asked participants the reasons why they stopped receiving care at a SAC: in the UK 2 reported having problems with travelling to the centre and 1 did not find the service useful. We only got one response in Germany; this person did not attend a SAC since 2018 and the annual review in 2020 was cancelled due to Covid. In Italy, out of 10 responses 2 reported problems with transport, 2 did not find the service useful, 3 found equal care locally and one stopped going because of transfer to another service (Supplementary Table 4).

For those who never went to a SAC in the UK, 3 said that their current level of care was sufficient and 4 said that the SAC are too far away to travel. In Italy, responses were split between 2 who did not wish to be referred, 2 who were not offered a referral and one person unsure of the reason (Supplementary Table 5; no data available for Germany). Among other reason why people did not attend a SAC, in the two countries people reported a lack of awareness about such referral pathway (see caption).

#### Access to an MDT

Regarding accessing an MDT clinic, 57.9% (*N* = 11) of respondents in the UK, 33% (*N* = 4) in Germany and 52.5% (*N* = 21) in Italy attended such clinic (Supplementary Tables 6a-c). We note a higher proportion of people attending MDT from SAC group in the UK and Italy compared to Germany. In Germany and Italy where participants completed the revised survey we asked who referred the participants to these MDT clinics: 3 were referred by their GP and one by the neurologist at general neurology clinic in Germany; in Italy, 8 (36.4%) were referred by the neurologist at general neurology clinic and 7 (31.8%) by neurologist at SAC. The responses from the remaining participants who attended an MDT clinic in that country (*N* = 7) showed the different ways to access an MDT (referrals from personal connections, physiatrist, physiotherapist, through AISA, and self-referral).

### Patient experience and satisfaction

Most patients (80%) in the UK, in Germany (75%) and in Italy (82%) who went to an MDT found the care received effective (Supplementary Tables 7 a-c). In the UK, we note a difference in feedback with all people in SAC and Used to SAC giving 100% positive feedback, whereas the feedback is split between 60% positive and 40% negative for the non-SAC group for the UK (*P* < 0.001). One person in that group who gave negative feedback said that there was no follow up treatment after the visit to MDT clinic (data not shown). In Germany the person who gave neutral feedback on the care received in MDT reported that the MDT did not understand their needs. In Italy, three people who gave neutral feedback commented saying that there was no treatment even after their visit to the MDT.

Looking specifically at the SAC services, all participants in SAC and Used to SAC provided positive feedback about their role in coordinating referrals, offering opportunity to take part in research studies, and securing parking badge in the UK (Table 4). The feedback was more mixed for their role in liaising with social workers and getting help to receive benefits. In Germany and Italy, the feedback was less positive for coordinating referrals and liaising with social workers, and quite positive for offers to take part in research (Table 4). In the revised survey for Germany and Italy, we asked specific questions in order to compare SAC and non-SAC services for some aspects of the healthcare services; results are summarised in Supplementary Tables 8a-h. Overall the feedback of people from Italy was positive for SAC services compared with non-SAC ones in terms of good understanding of the condition, helping people to cope better and live better with their condition including by providing medical advice on the management of their symptoms. The feedback from German participants was more mixed with only clear positive feedback for SAC compared with non-SAC services being for a good understanding of the condition.

**Table 4:** Participants’ feedback on SAC services.

% positive feedback	UK	Germany	Italy
Coordinating referrals to HCPs	100	46.1	51.4
Offers to take part in research	100	84.6	78.4
Liaising with social workers	66.7	58.3	48.7
Help with getting benefits	50	-	36
Help securing parking badge	100	-	-

Then we asked participants their feedback on various healthcare services they visited for their ataxia, by rating the three following statements: (1) HPCs understood how to manage my ataxia; (2) HCPs understood the treatment available for my ataxia. The participants’ feedback on their visits to primary care was mixed (Supplementary Tables 9a-f); as the level of knowledge and expertise increased (towards the specialist services from primary to secondary to tertiary care) so did the satisfaction of the patient visiting those services (Supplementary Tables 9g-r).

Overall, for the UK cohort of participants, feedback on the symptoms management was positive regardless of whether they were going to a SAC or not, however the care delivered in SAC was significantly more adapted to people needs than care in non-SAC (*p* < 0.001) (Supplementary Tables 10a, 11a). In Germany the SAC group feedback was positive (89%) for these two questions (Supplementary Tables 10b, 11b). For Italy the feedback about symptom management was 100% positive in non-SAC and 68% in SAC groups; the difference of positive feedback between these two groups was significant (*p* < 0.001). In that country, the feedback was different for the care delivered being adapted to people's needs with significant more positive feedback in SAC group (59%) compared to non-SAC (33%) (*p* < 0.001) (Supplementary Tables 10c and 11c). When we asked in the revised survey rolled out in Germany and Italy if the care people received in SAC was better than the care received in standard neurology clinic, 77% of participants in Germany and 51.5% in Italy answered positively.

### Patients’ feedback on how to improve the care

Across the three countries, the top three responses given by participants were more information about available treatment (UK: 17.1%; Italy 19.7%), followed by better access to therapies (UK: 13.2%; Germany: 11.5%; Italy: 15.6%) and better practical advice on living with FA (UK: 11.8%, Germany: 23.6%; Italy: 13.9%). We also had better management of symptoms and more information on help adapting the home for Germany as top responses (11.5%) (Supplementary Table 1^2^).

### Resource use and costs

In the UK the most common contacts for SAC patients (*N* = 3 patients) were other consultant specialist visits (mean 5.0 visits per patient per year) followed by occupational health therapy visits and physiotherapy visits (4.0 visits for each type) (Table 5). In Germany for SAC patients (*N* = 9 patients) these were physiotherapy visits (22.3) and occupational health therapy visits (15.6). In Italy for SAC patients (*N* = 25 patients) these were physiotherapy visits (19.0) and speech and language therapy visits (5.6). In the UK and Italy the differences in the numbers of contacts for all types of health service use between the SAC and non-SAC groups were mostly non-significant, except for the visit to other specialists in the UK where participants in SAC group had more visits than non-SAC group (no data for non-SAC group in Germany).

**Table 5:** Health care contacts and costs over a one-year period for non-SAC and SAC patients in three European countries.

Health care contacts	Patients who reported never attending a SAC				Patients who reported attending a SAC currently				Unit cost (euros)	*P*-value^a^	*P*-value^b^
	N	Mean	Std. Dev.	Median	N	Mean	Std. Dev.	Median			
**United Kingdom**											
Specialist centre visits	12	0	0	0	3	1.0	0	1	197	<0.01	<0.01
Family doctor visits	11	2.4	3.8	0	3	2.0	3.5	0	42	0.88	0.55
Neurologist visits	11	2.4	3.7	2	3	3.3	3.1	4	197	0.68	0.92
Inpatient stays	11	0.7	1.6	0	2	0	0	0	4,280	0.55	0.25
Emergency room visits	11	0.2	0.6	0	3	2.0	3.5	0	196	0.09	0.17
Physiotherapy visits	11	2.5	4.5	0	3	4.0	6.9	0	65	0.66	0.91
Speech and language therapy visits	11	0.4	1.2	0	3	0.7	1.2	0	185	0.70	0.17
Occupational health therapy visits	11	2.7	4.7	0	3	4.0	6.9	0	120	0.71	0.63
Other consultant specialist visits	11	0.2	0.6	0	3	5.0	5.3	3	119	<0.01	0.05
Total cost	10	4,664	8,394	827	2	829	725	829		0.55	0.26
**Italy**											
Specialist centre visits	3	0	0	0	25	1.2	0.4	1	39	<0.01	<0.01
Family doctor visits	3	0.3	0.6	0	20	0.4	0.7	0	28	0.97	0.73
Neurologist visits	3	0.7	0.6	1	20	0.6	0.9	0	29	0.90	0.72
Inpatient stays	3	0.3	0.6	0	21	0.2	0.4	0	5,575	0.74	0.77
Emergency room visits	2	0	0	0	21	0.1	0.2	0	29	0.77	0.79
Physiotherapy visits	3	23.3	23.0	10	23	19.0	23.2	2	25	0.77	0.99
Speech and language therapy visits	3	4.0	5.2	1	21	5.6	11.9	1	25	0.83	0.73
Occupational health therapy visits	2	5.0	7.1	5	19	1.4	4.6	0	25	0.33	0.58
Other consultant specialist visits	2	1.5	2.1	1	20	1.7	1.8	1	29	0.91	0.88
Total cost	2	958	449	958	14	963	1,533	139		0.99	0.78
**Germany**											
Specialist centre visits	0				9	1	0	1	170	-	-
Family doctor visits	0				9	5.3	3.7	4	29	-	-
Neurologist visits	0				9	1.9	1.7	2	65	-	-
Inpatient stays	0				8	0.6	1.2	0	4,950	-	-
Emergency room visits	0				9	2.1	4.9	0	144	-	-
Physiotherapy visits	0				9	22.3	26.2	1	38	-	-
Speech and language therapy visits	0				9	4.7	9.6	2	38	-	-
Occupational health therapy visits	0				9	15.6	21.3	2	38	-	-
Other consultant specialist visits	0				9	2.6	2.9	2	65	-	-
Total cost	0				8	5,604	6,031	2,820		-	-
***P*-value** ^c^											
Specialist centre visits	-				0.64						
Family doctor visits	0.81				0.36						
Neurologist outpatient visits	0.83				0.03						
Inpatient stays	0.72				0.98						
Emergency room visits	-				0.90						
Physiotherapy visits	0.02				0.13						
Speech and Language therapy visits	0.08				0.55						
Occupational Health Therapy visits	0.52				0.73						
Other consultant specialist visits	0.19				0.42						
Total cost	0.91				0.87						

a Test for significant differences in mean contacts and total costs between Non-SAC and SAC groups (unadjusted).

b Test for significant differences in mean contacts and total costs between Non-SAC and SAC groups (adjusted for age, sex, number of symptoms and number of comorbidities).

c Test for significant differences in mean contacts and total costs between countries separately for Non-SAC and SAC groups (adjusted for age, sex, number of symptoms and number of comorbidities).

Unit costs and total costs are in 2023 €.

SAC, specialist ataxia centre; N, number of participants who responded to that question.

For the non-SAC group there were differences between countries in terms of physiotherapy visits (the highest number of mean visits per patient per year were in Italy, followed by the UK, *P* = 0.02). For the SAC group there were significant differences between countries in terms of neurologist outpatient visits (the highest number of visits were in the UK, then Germany, then Italy, *P* = 0.02).

There were no statistical differences in mean total costs per patient per year between the non-SAC and SAC groups in the UK and Italy (data not available for non-SAC group in Germany). In the UK, the costs are 4664 euros for the non-SAC group and 829 euros for the SAC group; it is important to note that inpatient stay reported in non-SAC participants did contribute to this higher cost. The mean total cost per patient per year for SAC group in Germany is 5604 euros; again, here the inpatient stay reported contributed to this higher cost compared to the other SAC groups. For Italy, the costs are 958 euros per patient per year for non-SAC group vs. 963 euros for the SAC group. We did not find any statistical differences in these costs between countries in both the non-SAC and SAC groups (Table 5).

### Patient travel

Noting the small numbers of respondents, among those who attended the SAC, the modal travel time was 1 to 2 h in the UK and Italy and less than 1 h in Germany (Table 6). A small proportion of patients travelled for more than 2 h in all three countries (33% in the UK; 16% in Germany; 30% in Italy). The most common mode of transport to the SAC was via car in all three countries, and the second most common was via train in all three countries.

**Table 6:** Mode of transport and time taken to travel to the SAC in three European countries.

Travel time to visit the Specialist Ataxia Centre (one way)			Mode of transport mainly used to visit the SAC		
Travel time	N	%	Mode of transport	N	%
**UK (*N*** **=** **3)**					
Less than 1 h			Bus		
1 to 2 h	2	67	Car	2	67
2 to 3 h			Taxi		
3 to 4 h	1	33	Train	1	33
More than 4 h			Other		
Unsure			Unsure		
**Italy (*N*** **=** **30)**					
Less than 1 h	10	33	Bus		
1 to 2 h	11	37	Car	27	90
2 to 3 h	3	10	Taxi		
3 to 4 h	4	13	Train	3	10
More than 4 h	2	7	Other		
Unsure	0	0	Unsure		
**Germany (*N*** **=** **13)**					
Less than 1 h	9	69	Bus		
1 to 2 h	2	15	Car	7	54
2 to 3 h	1	8	Taxi	1	8
3 to 4 h	1	8	Train	4	31
More than 4 h			Other	1	8
Unsure			Unsure		

For both Non-SAC, and SAC patients the modal travel time to see the neurologist at the general neurology clinic was less than 1 h in all three countries where these data were available (Supplementary Table 1^3^). In Italy the majority of patients travelled to see the neurologist at the general neurology clinic by car.

## Discussion

The aim of this study was to get a better understanding of the patient pathway of people living with Friedreich's ataxia in different European countries, focusing on the journey to get a diagnosis followed by treatment and care in order to manage the condition. The patient survey we rolled out in the UK, and revised version in Germany and Italy, allowed us to gather crucial information about patient's resource use, experience and feedback of their visits to various healthcare services including SACs. Ultimately, we wanted to evaluate the impact of visiting SAC services vs. non-specialist neurology clinics on patients’ symptoms management and quality of care delivered to people living with FA together with costs associated.

### Participants living circumstances

The cohort of participants across the three countries has lived with FA for some time, evidenced by their diagnosis being reached for a majority more than five years ago. Most people reported being affected progressively by their ataxia for their daily activities for several years to decades. A majority of participants across the three countries lives with a spouse/family members or a friend, whom 72% respondents in Italy identified this person being an unpaid carer. These results suggest that in the cohort we have, the possibility to live independently, without needing assistance for daily activities is limited. In addition, 72% of participants in the UK are not working (never worked or took early retirement) which can lead to a situation of financial dependency. Considering only a small proportion of people reported receiving welfare support, this can lead to serious challenges in affording adapted care at home, for example when there's a need to pay for a carer. A higher proportion of people in Germany and Italy remained at work. In a recent study on progressive ataxias (19) we have found a strong association between employment status and attendance to SAC, with a higher proportion of people attending a SAC working compared to people who stopped going to a SAC. The higher proportion of people in SAC group in Italy and Germany compared to the UK could explain that difference.

### Reaching a diagnosis

Friedreich's ataxia diagnosis remains challenging to diagnose early and with an onset usually at puberty this can lead to delay in the specialised care (20). Considering the majority of participants got their diagnosis from a neurologist, the pathway to be referred to such HCP is crucial for a timely diagnosis and the elaboration of a care plan. A faster referral to a neurologist was reported in Germany and Italy compared to the UK. Interestingly half of participants in Italy went straight to a SAC for that referral to see a neurologist vs. 15% in Germany. This might explain how 60% of people in Italy got their diagnosis in a SAC vs. 37% in Germany. However, in these two countries, the data collected with our survey doesn't allow us to conclude on the role of SACs in this cohort in terms of reaching a diagnosis timely. Further research would be needed to assess the role of such centres in the time to reach a diagnosis.

### Access to a SAC

The referral pathway to attend a SAC shows similarities between the countries with people being referred primarily by the neurologist (non-specialist clinic) and secondly by the GP. However, in Germany and Italy we observed a proportion of people who accessed a SAC through self-referral, and referral from other HCPs. We noted a mixed referral pathway in these two countries for progressive ataxias, highlighting a different use of SACs by patients who might be able to self-refer themselves and the lack of recognition by National Health systems for such centres evidenced by a lack for formal referral pathways (21, 22).

Transport ranked high in the UK and Italy as a reason why stopped going to a SAC, followed by finding the service not useful and finding current level of care sufficient. We note that the travel to SAC in Germany tends to be shorter compared to the UK and Italy. In comparison with that journey, the travel time for people to go to a locally neurologist is shorter in both the UK and Italy, and similar to Germany. So, a longer travel to SAC might lead to more physical challenges to access a centre and more fatigue. This is a significant barrier for people living with FA as in the top 5 of patient-reported most prevalent and impactful symptoms we can find impaired coordination, limitations with mobility and walking, inability to do activities, fatigue and lower extremity weakness (23). In the UK and Italy, participants who never visited a SAC reported a lack of awareness about the centres and the fact of not being offered a referral. Travelling was also a top reason why people in the UK did not attend a SAC. This result is supported by a previous study where we discussed the low number of centres across the UK, all concentrated in England, which suggests inequality in accessing a specialist centre and highlights the need for more centres in the UK for the care of people with ataxia (24). Whereas in Italy we find more centres in different parts of the country. This suggests that the number of centres is not enough to facilitate patients’ attendance and adherence to SAC services. A combination of telemedicine and in-person attendance at SAC services could be a solution to maintain ataxia patients’ care under SAC.

### Access to an MDT

A significant proportion of participants attended an MDT clinic in the UK and Italy compared to Germany. This was also the case when we looked at all progressive ataxias (14) although for each country there's a higher proportion of FA participants who accessed an MDT. The place of MDT within the care model is different between these countries, with the UK first adopted the MDT approach within the care model in SAC (10, 11) and then Italy with professionals running such MDT clinics usually based at the hospital where the SAC is (22). The situation in Germany is different as SACs are not based on that model; they can facilitate access to MDT clinics without being reimbursed in the SAC outpatient setting (21). In Germany, the GP primarily referred people to such clinic whereas in Italy no one was referred by a GP; indeed, in this country the referrals were coming from the neurologist at a SAC, the non-SAC neurologist and other pathways (self-referrals, charity, other HCPs). So, the pathway to access an MDT is different between Italy and Germany with surprisingly no referral coming from neurologists in SAC in Germany, as our Germany cohort is made of people currently attending a SAC. This was also the case for the study on all progressive ataxias in that country where no referral was made by neurologist at a SAC (21). This result is supported by the participants feedback about the role of SAC services in Germany with only 46.5% being positive on their role in coordinating referrals to other HCPs.

### Patient experience and satisfaction

For most participants of the three countries, the MDT care was effective. For the few people who gave neutral to negative feedback, they reported a lack of follow up treatment after their MDT visit. Overall, the care delivered by MDT can make a difference to FA patients attending and this highlights the need to increase the accessibility to such clinics and integrate them into the care provision by SAC as we previously recommended for progressive ataxias through the SAC (14). Such clinic being part of the SAC service could make a difference in terms of follow up treatment after the visit to the MDT clinic.

In terms of feedback about aspects of SAC service, attending a SAC helped participants to take part in research. It is crucial that such centres run translational research so future treatments will be more evidence based (14). Centres in the UK clearly helped in coordinating referrals to other HCPs of participants; on the other hand, in Germany and Italy around half of the people who attended a SAC were positive about this aspect. For the liaison with social workers, again the feedback was more mixed particularly in Germany and Italy, so developing further this role of the SAC would be beneficial for the support people can receive when being at home. These results show that the SACs in the UK play a significant role for the coordination of the care after the visit to SAC compared with Germany and Italy where the clinical research seems to be the primary focus. This reinforces to need for more implication of SAC services in the disease management in these two countries (14, 21). Across the three countries, the connection between the SACs services and the social services could be developed further in order to support people in their life at home, particularly financially, and when adaptation to the home is required.

As expected, the patients feedback was more positive about the level of understanding of their condition, management and treatment available as they saw HCPs with more knowledge and expertise, with best feedback being for their visit to SAC, followed by secondary care services, and mixed feedback from primary care services. The level of understanding of FA was recognised by participants attending SAC in Italy with a more personalised care being delivered in such centres compared to non-SAC services. However, in this country, people felt the management of their symptoms was significantly less good in the specialist centres. In this context, only 51.5% answered positively regarding the overall care received being better in SAC vs. non-SAC. We can link that feedback to the fact that 50% people in Italy who stopped going to SAC found the service not useful and the current level of care they received elsewhere was sufficient. This mixed feedback from people in Italy attending SACs supports the suggestion we made above that these centres need more implications in disease management alongside the personalised care approach.

Overall, the care delivered in SAC was more adapted to peoples’ needs in the UK and in Italy compared to care delivered in non-SAC. In addition, 77% participants from Germany felt the care delivered in SAC was better than non-specialist clinics. These results suggest that the SAC services are able to deliver a more personalised care based on patients’ needs compared to non-specialist clinics.

### Resource use and costs associated

The health economic evaluation showed no difference in HCPs visits between SAC and non-SAC groups for the UK and Italy, and the costs per patient per year was not significantly different between these groups. In the context of patients’ feedback provided on SAC vs. non-SAC, our results highlight the patient's appreciation for SAC services compared to non-SAC without any significant change in costs. In the UK, participants in SAC group had more visits to other specialists for their ataxia than those in non-SAC group; this might be a consequence of the SAC in UK being better at coordinating referrals than in non-SAC settings. Looking specifically at SAC groups, participants in the UK reported more outpatient neurologist visits compared to Germany and Italy. This observation is supported by a previous study on the impact of FA on HC resource use showing the top HCPs visits in the UK being the neurologist (25). In non-SAC groups, the fact that people in Italy attended more physiotherapy visits suggest a better support towards management of symptoms outside the SAC and aligns with the health system in Italy which allows people to go more often to such HCPs (communication with focus group in Italy). There was no significant difference in costs for SAC and non-SAC groups between countries, despite the costs of non-SAC group in the UK and SAC group in Germany being more expensive than in the two other countries. The variation in costs being large and the mean differences being not statistically significant was also observed in our previous studies looking at all progressive ataxias (21, 24), implying that small sample sizes, especially in the analyses by subgroups impacted the cost analysis outcomes.

Previous studies on FA costs have highlighted the challenge to accurately evaluate the economic burden of the disease (25, 26), considering all aspects of care utilisation and costs associated, as well as cost to society when it comes to living with a progressive condition for decades. More work is needed to fully estimate the true economic burden of FA, and to assess the impact of attending a SAC on health outcomes, quality of life and other costs associated (out-of-pocket expenses, time off work), to inform where to allocate more resources to treat FA patients efficiently.

### Limitations of this study

Recruitment through patient advocacy organisations may have introduced participation bias, as respondents are more likely to be proactive, informed, and engaged with specialist services than the broader FA population. This may limit the generalisability of findings and is important to consider when interpreting cross-country comparisons. The fact we don't have participants in the non-SAC group on Germany prevented us from running any comparison test and stats between SAC and non-SAC groups in this country. The cohort in Germany is small compared to the two other countries and all people recruited living with FA have been to a SAC as part of their care. We also note that once we stratified the UK and Italy cohort by SAC attendance, we have a small group of SAC participants and non-SAC participants in the UK and Italy respectively. Resource use data for Italy and Germany were collected for the preceding 12 months, whereas for the UK they were only collected for the previous 6 months. To make the figures more comparable we multiplied the UK figures by two (16). We acknowledge this might not completely reflect 12 months resource use in the UK; however, we also compared our survey data with data from clinical practice in the two UK specialist ataxia centres and the numbers were broadly consistent.

## Conclusion and recommendations

The survey has highlighted patients’ appreciation for SAC services compared to non-SAC, without any significant increase in the costs, with the ability to deliver a more personalised care based on patients’ needs compared to non-specialist clinics. We identified difficulties in patients’ attendance and adherence, being physical distance to centres, and a lack of awareness about SACs. Having a clear and standardised referral pathways to HC services where expertise in rare diseases like FA can be found, together with education programmes on such diseases in primary care and local hospital clinicians, would facilitate the treatment and care of these patients (27). Telehealth in conjunction with primary care teams has the potential to transform SAC accessibility, particularly for those with a greater disease burden, and future work should explore applicability with this particular disease group (19). Our study shows that the visit to an MDT clinic was effective for FA patients. Yet the access to MDT clinics could be improved with incorporating these into the SAC services like in the UK. The presence of MDT as part of the SAC model in the UK was established twenty years ago when the first multidisciplinary centre was open (10, 11). Across the three countries, participants reported that what matters the most to them is getting more information about available treatment, better access to therapies and better practical advice on living with FA. These would contribute to a better management of symptoms and ability to live a better life after healthcare services visits.

## Data Availability

The raw data supporting the conclusions of this article will be made available by the authors, without undue reservation.
