# Supplementary material for: Friedreich's ataxia patient pathway in Europe
Source: Front Health Serv. 2026 May 28;6:1817584. doi: 10.3389/frhs.2026.1817584 (PMC13254176; doi:10.3389/frhs.2026.1817584)
Supplement: Supplementary file 3 [file Supplementaryfile3.docx]

**APPENDIX 3: Ataxia Care Pathways – Italy Patient Survey**

**Patient Care Pathways Survey**

Thank you for taking the time to complete and submit this survey. Please take time to read the participant information sheet provided alongside this survey carefully, and feel free to discuss it with others, such as your family and friends.

The purpose of this study is to gather the experiences of care of people with ataxia in Italy, in order to understand ataxia patients’ care pathways and history of accessing specialist ataxia centres in Italy.

You may wish to discuss your answers to the survey with your parents if you were diagnosed when you were very young. Parents/carers/guardians filling in the survey should respond as per the patient’s opinion, not your own.

**Personal details and confidentiality**

All the information you provide during the course of this study will be kept confidential. No one participating in the study will be able to identify you based on the answers, unless you indicate that you want to be contacted for future studies or want to receive a copy of the study by email at the end.

Your survey responses will be combined with responses from other patients in the study, to allow analyses to be carried out by the appointed research team. This way, it will not be possible to identify any single patient from the pooled survey response data. All data will be stored in a form protected by passwords so only people working on the study will be able to access it, and will be deleted 6 months after study analyses are complete.

**Organisation and funding of the study**

This study has been sponsored by the European Brain Council (EBC), is funded by Takeda Pharmaceutical Company Ltd and Reata Pharmaceuticals Inc and is being carried out by the charity AISA and University College London.

**Contact information**

Should you wish to ask any further questions about the study before, during or after it has been completed, or if you have any concerns, please contact

Julie Vallortigara

Email : j.vallortigara@ucl.ac.uk

**Completing the study**

Please fill the survey online if possible by answering all questions, unless indicated otherwise, by ticking the relevant box or by writing your answer.

**Definitions of specific terms used in the survey are provided below**

**Medical terms and definition**

General ataxia diagnosis: the first time that a healthcare provider told you that you had ataxia.

Specific ataxia diagnosis particular type of ataxia, e.g. spinocerebellar ataxia type 1: when a healthcare provider told you that you that you had a. Some people completing this survey will not have a specific ataxia diagnosis.

For the purposes of this survey, idiopathic ataxia syndrome and cerebellar ataxia (unknown cause) are not considered specific diagnoses.

Specialist ataxia centre (SAC): a centre that specialises in care of ataxia patients only.

List of SACs in Italy and the clinicians working in each of these centres:

- FIRENZE, Clinica Neurologica, Policlinico, Università degli Studi; DOTT.SSA PIACENTINI SILVIA sostituita da Dott.ssa Camilla Ferrari

- MILANO, IRCCS, Istituito Neurologico “C. Besta”; DOTT.SSA MARIOTTI CATERINA

E DOTT. TARONI FRANCO

- MESSINA, Policlinico, Università degli Studi; DOTT.SSA MUSUMECI OLIMPIA

- NAPOLI, AOU Federico II, Clinica Neurologica, Università degli Studi; PROF FILLA ALESSANDRO e PROF SACCA’ FRANCESCO

- ROMA, Università degli Studi, La Sapienza, Polo Latina; DOTT. CASALI CARLO

- ROMA, IRCCS, Bambin Gesù; DOTT. BERTINI ENRICO

- SIENA, Policlinico, Le Scotte, Università degli Studi; Prof. ANTONIO FEDERICO

- TORINO, SC Neurologia- AOU Città della Salute e della Scienza di Torino; DOTT. BRUSCO ALFREDO e DOTT.SSA ORSI LAURA

- PISA IRCCS Stella Maris PROF. SANTORELLI FILIPPO

- GENOVA IRCCS Ospedale Policlinico San Martino DOTT. FANCELLU ROBERTO

- BOLOGNA Centro il BeNe Dott. SALVI FABRIZIO

There are other doctors around the country with an interest in ataxia, but this survey is specifically considering specialist ataxia centres, and these doctors should not be considered as specialist ataxia centres.

General Neurology clinic: neurology service within an hospital with generalist clinic and not specialised on ataxia.

Primary care: Healthcare delivered outside hospitals. It includes a range of services, including community clinics, health centres and walk-in centres, and is delivered by primary care professionals (please see below).

Primary care professional: someone who provides medical care outside of a hospital, e.g. a general practitioner (GP), nurse practitioner, pharmacist, health visitor, midwife, dentist, or optician.

Secondary care: Healthcare provided in hospitals. This includes accident and emergency departments, outpatient departments, antenatal services, genitourinary medicine and sexual health clinics.

Inpatient stay: an overnight admission to a hospital.

Outpatient visit: a visit to a hospital to undergo tests or receive same-day surgery.

Tertiary care: Care for people needing complex treatments. People may be referred for tertiary care (for example, a specialist stroke unit) from either primary care or secondary care. Tertiary referral centres are usually large hospitals which provide inpatient care (i.e. where you are admitted overnight).

Primary healthcare contact: the person you would contact first if you had queries about your condition or treatment.

MDT clinic: Multidisciplinary teams include more than one of the following people as part of the same service (in the same clinic): physiotherapists, occupational therapists, orthotics or speech and language therapists.

**SURVEY**

**1) General / demographic questions**

Question 1 – mandatory to continue the survey

I confirm that I am (or the person I am completing this survey on behalf of is) aged 16 years or over, live(s) in the UK and wish(es) to proceed with this survey.

PLEASE CHECK ONE:

• Yes

• No

Question 2 (first question of the survey after giving consent)

Please indicate whether you are the person living with ataxia or a person completing the survey on their behalf and representing patient’s view

PLEASE CHECK ONE:

• Yes, I am the person living with ataxia

• No, I am completing it on behalf of the person with ataxia

Question 3

Have you or the person you are completing the survey on behalf of been diagnosed with ataxia by a healthcare professional?

PLEASE CHECK ONE:

• Yes

• No [Please discontinue the survey]

• Unsure [Please discontinue the survey]

Question 4

What is your age? (patient age, as a note that pops up on the platform)

PLEASE CHECK ONE:

•16–29 •30–59 •60–80 •80+

Question 5

Which of the following best describes your gender? (patient gender, as a note that pops up on the platform)

PLEASE CHECK ONE:

•Female •Male •Not listed

Question 6

Do you have any additional conditions, not related to your ataxia?

PLEASE CHECK ALL THAT APPLY:

• Diabetes mellitus

• Liver disease

• Malignancy (cancer)

• HIV or AIDS

• Chronic kidney disease

• Congestive heart failure

• Myocardial infarction (heart attack)

• Chronic obstructive pulmonary disease

• Peripheral vascular disease

• Cerebrovascular accident or transient ischemic attack (stroke)

• Dementia or Alzheimer’s disease

• Psychiatric disorder (depression or any other)

• Hemiplegia (paralysis of one side of the body)

• Rheumatic or connective tissue disease

• Peptic (stomach) ulcer disease

• None

• Other [Please specify in the box below]

• Not sure

TEXT BOX

Please enter your response here

Question 7

Do you live alone?

PLEASE CHECK ALL THAT APPLY:

• Yes, I live alone

• No, I live with a spouse or with family members and I do not require assistance that significantly limits their work inside or outside home.

• No, I live with a carer and my carer is a professional

• No, I live with a carer and my carer is a not a professional (friends, partner, family)

Question 8

Which geographical region of the UK do you live in?

PLEASE CHECK ONE:

• Piemonte

• Trentino Alto Adige

• Veneto

• Liguria

• Toscana

• Marche

• Lazio

• Emilia Romagna

• Umbria

• Basilicata

• Campania

• Calabria

• Sicilia

• Val d’Aosta

• Lombardia

• Friuli Venezia Giulia

• Abruzzo

• Molise

• Puglia

• Sardegna

Other [Please specify in the box below]

• Unsure

TEXT BOX

Please enter your response here

Question 9

Which of the following best describes how your ataxia affects your mobility?

PLEASE CHECK ONE:

• No functional impairment

•. Mild, able to run, walking unlimited

•. Moderate, unable to run, limited walking without aid

•. Walking with one stick

•. Walking with two sticks/aid of walker indoors, utilising wheelchair outdoors

•. Unable to walk, requiring wheelchair, still independent

•. Unable to walk, wheelchair bound, dependent

•. Confined to bed

Question 10

How has your ataxia affected your working life?

PLEASE CHECK ALL THAT APPLY:

• I am still in education

• I still work

• I have retired

• I have never worked because of my ataxia

• I have taken early retirement because of my ataxia [Please specify the age of retirement in the box below]

• I have changed jobs because of my ataxia

• I have changed to working fewer hours or part-time because of my ataxia

• I have received welfare support because of my ataxia

• I have received financial support from my family because of my ataxia

• I have taken sick leave because of my ataxia [Please specify how many days per year in the box below]

• Other [Please specify in the box below]

TEXT BOX

Please enter your response here

Question 11

Which health professional is currently responsible for your ataxia management?

PLEASE CHECK ONE:

• General practitioner (GP)

• Neurologist in a general neurology clinic (not at a specialist ataxia centre)

• Neurologist at a specialist ataxia centre

• Other [Please specify in the box below]

• Unsure

TEXT BOX

Please enter your response here

**2) Diagnosis questions**

Question 12

Which ataxia do you have?

PLEASE CHECK ONE:

• Friedreich’s ataxia

• Inherited cerebellar ataxia (SCAs e.g. SCA1, SCA2, SCA3, etc.)

• Cerebellar ataxia (unknown cause and not identified by genetic testing)

• Episodic ataxia

• Other [Please specify in the box below]

• Not known

• none [Please discontinue the survey]

TEXT BOX

Please enter your response here

Question 13

Which healthcare professional gave you the first general diagnosis of ataxia?

PLEASE CHECK ONE:

• General practitioner (GP)

• Neurologist

• Physiotherapist

• Geneticist

• Other [Please specify in the box below]

• Unsure

TEXT BOX

Please enter your response here

Question 14

When this healthcare professional told you this first general diagnosis of ataxia?

TEXT BOX

Please enter your response here

MM (if known) and YYYY

• Unsure

Question 15

Has your specific diagnosis of ataxia been confirmed e.g. through genetic testing?

PLEASE CHECK ONE:

• Yes, I have a genetic confirmation of my diagnosis e.g. Friedreich’s ataxia, spinocerebellar ataxia type 1, 2, 3, etc. (SCA1, SCA2, SCA3, etc.) [Please specify in the box below]

• Yes, I have other confirmation of my diagnosis e.g. gluten ataxia [Please specify in the box below]

• No

• Unsure

TEXT BOX

Please enter your response here

Question 16

Where did you get confirmation of your specific diagnosis (genetic or other ataxia i.e. gluten/paraneoplastic ataxia or others)?

PLEASE CHECK ONE:

• In a specialist ataxia centre

• In a general neurology clinic (non-specialist service)

• Other [Please specify in the box below]

• Unsure

TEXT BOX

Please enter your response here

Question 17

How long did it take from being first seen by a neurologist to when you received a confirmed specific ataxia diagnosis, if you have received one (e.g. Friedreich’s ataxia, spinocerebellar ataxia type 1 etc.)?

PLEASE CHECK ONE:

• I received the specific ataxia diagnosis at the time I was seen by the neurologist for my ataxia

• Up to 6 months

• Between 6 months and 1 year

• Between 1 and 2 years

• Between 2 and 5 years

• More than 5 years

• I have not received a specific ataxia diagnosis [please proceed to question 19]

• Unsure

Question 18

How many times were you told by a healthcare professional that you might have a different condition or symptom, e.g. multiple sclerosis, dyspraxia, apraxia, ear problems, vertigo, etc.), before you received your specific ataxia diagnosis?

PLEASE CHECK ONE:

• None

• 1

• 2

• 3

• 4

• 5 or more

• Unsure

Question 19

When you received your first general ataxia diagnosis, how much were your activities of daily living affected by your ataxia?

PLEASE CHECK ONE:

• My ataxia did not affect me

• My ataxia caused occasional problems

• My ataxia caused frequent problems restricting my activities

• My ataxia caused constant problems that restricted me most or all of the time

• Unsure

Question 20

How much are your activities of daily living affected by your ataxia now?

PLEASE CHECK ONE:

• My ataxia does not affect me

• My ataxia causes occasional problems

• My ataxia causes frequent problems restricting my activities

• My ataxia causes constant problems that restricted me most or all of the time

• Unsure

Question 21

If you have a genetic diagnosis, have you discussed the implications on insurance or family planning with a healthcare professional?

PLEASE CHECK ONE:

• Yes

• No

• N/A

• Unsure

**3) Referrals and appointments**

Question 22a

How long did it take from the first time you sought medical advice for your first symptom of ataxia (e.g. visited a GP to discuss your symptom) to the point at which you were referred to a neurologist?

PLEASE CHECK ONE:

• I was referred to a neurologist immediately

• Up to 6 months

• Between 6 months and 1 year

• Between 1 and 2 years

• Between 2 and 5 years

• More than 5 years

• Unsure

Question 22b

Where was the neurologist you were referred to:

PLEASE CHECK ONE:

• In a specialist ataxia centre

• In a general neurology clinic (non-specialist service)

• Other [Please specify in the box below]

• Unsure

TEXT BOX

Please enter your response here

Question 23a

How many of the following types of appointment did you attend before your first (general) diagnosis of ataxia?

PLEASE CHECK ONE PER LINE:

GP appointments

• None •1 • 2 to 3 • 4 to 5 • More than 5

• Cannot recall

Hospital outpatient clinic visits with a neurologist (not at a specialist ataxia centre)

• None •1 • 2 to 3 • 4 to 5 • More than 5

• Cannot recall

Specialist ataxia centre visits

• None •1 • 2 to 3 • 4 to 5 • More than 5

• Cannot recall

Hospital inpatient stays: how many times

• None •1 • 2 to 3 • 4 to 5 • More than 5

• Cannot recall

Hospital inpatient stays: how many nights

• None •1 • 2 to 3 • 4 to 5 • More than 5

• Cannot recall

Accident and Emergency visits

• None •1 • 2 to 3 • 4 to 5 • More than 5

• Cannot recall

Physiotherapist appointments

• None •1 • 2 to 3 • 4 to 5 • More than 5

• Cannot recall

Speech and language therapist appointments

• None •1 • 2 to 3 • 4 to 5 • More than 5

• Cannot recall

Occupational therapist appointments

• None •1 • 2 to 3 • 4 to 5 • More than 5

• Cannot recall

Other consultant specialists (e.g. an ophthalmologist or an ear, nose and throat [ENT] specialist [Please specify the consultant in the box below]

• None •1 • 2 to 3 • 4 to 5 • More than 5

• Cannot recall

TEXT BOX

Please enter your response here

Question 23b

How many of the following types of appointment did you attend before your specific diagnosis of ataxia?

PLEASE CHECK ONE PER LINE:

GP appointments

• None •1 • 2 to 3 • 4 to 5 • More than 5

• Cannot recall

Hospital outpatient clinic visits with a neurologist (not at a specialist ataxia centre)

• None •1 • 2 to 3 • 4 to 5 • More than 5

• Cannot recall

Specialist ataxia centre visits

• None •1 • 2 to 3 • 4 to 5 • More than 5

• Cannot recall

Hospital inpatient stays: how many times

• None •1 • 2 to 3 • 4 to 5 • More than 5

• Cannot recall

Hospital inpatient stays: how many nights

• None •1 • 2 to 3 • 4 to 5 • More than 5

• Cannot recall

Accident and Emergency visits

• None •1 • 2 to 3 • 4 to 5 • More than 5

• Cannot recall

Physiotherapist appointments

• None •1 • 2 to 3 • 4 to 5 • More than 5

• Cannot recall

Speech and language therapist appointments

• None •1 • 2 to 3 • 4 to 5 • More than 5

• Cannot recall

Occupational therapist appointments

• None •1 • 2 to 3 • 4 to 5 • More than 5

• Cannot recall

Other consultant specialists (e.g. an ophthalmologist or an ear, nose and throat [ENT] specialist [Please specify the consultant in the box below]

• None •1 • 2 to 3 • 4 to 5 • More than 5

• Cannot recall

TEXT BOX

Please enter your response here

Question 24

In THE PAST YEAR, how many of the following types of appointment have you attended as a result of your ataxia?

PLEASE CHECK ONE PER LINE:

GP appointments

• None • 1 • 2 to 3 • 4 to 5 • More than 5

• Cannot recall

Hospital outpatient clinic visits with a neurologist (not at a specialist ataxia centre)

• None • 1 • 2 to 3 • 4 to 5 • More than 5

• Cannot recall

Specialist ataxia centre visits

• None • 1 • 2 to 3 • 4 to 5 • More than 5

• Cannot recall

Hospital inpatient stays: how many times

• None • 1 • 2 to 3 • 4 to 5 • More than 5

• Cannot recall

Hospital inpatient stays: how many nights

• None • 1 • 2 to 3 • 4 to 5 • More than 5

• Cannot recall

Accident & Emergency visits

• None • 1 • 2 to 3 • 4 to 5 • More than 5

• Cannot recall

Physiotherapist appointments

• None • 1 • 2 to 3 • 4 to 5 • More than 5

• Cannot recall

Speech and language therapist appointments

• None • 1 • 2 to 3 • 4 to 5 • More than 5

• Cannot recall

Occupational therapist appointments

• None • 1 • 2 to 3 • 4 to 5 • More than 5

• Cannot recall

Other consultant specialists (e.g. an ophthalmologist or an ear, nose and throat [ENT] specialist) [Please specify the consultant in the box below]

None 1 2 to 3 4 to 5 More than 5

Cannot recall

Question 25

Have you ever been referred to/seen at one of the eleven specialist ataxia centres (Florence, Milan, Messina, Naples, Rome (2 centres), Siena, Turin, Pisa, Genova, Bologna)?

PLEASE CHECK ONE:

􀂅 Yes, I currently receive care at a specialist ataxia centre

􀂅 Yes, I was seen at a specialist ataxia centre but no longer receive care there

􀂅 No [please proceed to question 25i]

􀂅 Unsure [please proceed to question 25i]

Question 25a

Before being referred to the specialist ataxia centre, have you been referred / seen as a patient in a general neurology clinic (non-specialist clinic) for your ataxia?

PLEASE CHECK ONE:

􀂅 Yes

􀂅 No

􀂅 Unsure

Question 25b

When were you referred to the specialist ataxia centre?

TEXT BOX

Please enter your response here

MM (if known) and YYYY

• Unsure

Question 25c

Who referred you?

PLEASE CHECK ONE:

• GP

• Other primary care professional

• Hospital neurologist

• Other [Please specify in the box below]

• Unsure

TEXT BOX

Please enter your response here

Question 25d

If you have previously attended other neurological clinics for your condition, before attending a specialist ataxia centre, do you feel the care received at the specialist ataxia centre is an improvement of the care you previously received?

PLEASE CHECK ONE:

• Yes

• No

• N/A

• Unsure

Please compare your experience at the specialist ataxia centre with the general neurology clinic (non-specialist clinic) by CHECKING ONE BOX PER ROW FOR EACH OF THE FOLLOWING STATEMENTS.

Only complete this question if you attended both specialist ataxia centre and non-specialist clinic. Otherwise skip to question 25.

Please tick one option per row if applicable

Very much better at the specialist ataxia centre

Better at the specialist ataxia centre

Neither better nor worse at the specialist ataxia centre

Worse at the specialist ataxia centre

Much worse at the specialist ataxia centre

A good understanding of your condition

Makes you able to cope better with your condition

Gives practical advice about how to live better with my condition

Provides medical advice on the management of your symptoms

Coordinating onward referral to specialists

Offers to participate in research

Help with benefits

Improved communication between health and social care professionals about my needs

Question 25e

If you no longer receive care at a specialist ataxia centre why is that?

PLEASE CHECK ONE:

• Problems with travelling/transport

• Did not find it useful

• Not referred again

• Equal care locally

• Unable to take time off work to visit the centre

• Other [Please specify in the box below]

• Unsure

TEXT BOX

Please enter your response here

Question 25f

How long does/did it take you to travel to visit the specialist ataxia centre (one way)?

PLEASE CHECK ONE:

• Less than 1 hour

• 1 to 2 hours

• 2 to 3 hours

• 3 to 4 hours

• More than 4 hours

• Unsure

Question 25g

What is the main method of transport that you usually use/used to travel to the specialist ataxia centre?

PLEASE CHECK ONE:

• NHS transport

• Car

• Bus

• Train

• Taxi

• Walk

• Other [Please specify in the box below]

• Unsure

TEXT BOX

Please enter your response here

Question 25h

Do you/did you need overnight accommodation when you travel/travelled to the specialist ataxia centre?

PLEASE CHECK ONE:

􀂅 Yes, it was hospital accommodation

􀂅 Yes, it was paid for privately

􀂅 No

􀂅 Unsure

Question 25i

If you have never been referred to/seen at a specialist ataxia centre, why is this?

PLEASE CHECK ONE:

􀂅 Current level of care is sufficient

􀂅 I asked to be referred to a specialist ataxia centre but was refused by my doctor

􀂅 The specialist ataxia centres are too far away for me to travel to

􀂅 Did not wish to be referred

􀂅 A referral was not offered

􀂅 Other [Please specify in the box below]

􀂅 Not applicable

􀂅 Unsure

TEXT BOX

Please enter your response here

Question 26

How long does/did it take you to travel one-way to receive ataxia care by a neurologist that is base in general neurology clinic (not at a specialist ataxia centre)?

PLEASE CHECK ONE:

􀂅 Less than 1 hour

􀂅 1 to 2 hours

􀂅 2 to 3 hours

􀂅 3 to 4 hours

􀂅 More than 4 hours

􀂅 Not applicable

􀂅 Unsure

**4) Level of care received and satisfaction**

Question 27

Have you ever been seen by a multidisciplinary team (MDT), including at a non-specialist hospital?

PLEASE CHECK ONE:

• Yes

• No [please proceed to question 28]

• Unsure [please proceed to question 28]

Question 27a

Who referred you to the MDT clinic:

PLEASE CHECK ONE:

• Neurologist in a specialist ataxia centre

• Neurologist in a general neurology clinic (non-specialist service)

• Other [Please specify in the box below]

TEXT BOX

Please enter your response here

Question 27b

When were you referred to the MDT clinic?

TEXT BOX

Please enter your response here

MM (if known) and YYYY

• Unsure

Question 27c

If you have been seen by a multidisciplinary team, including at a non-specialist hospital, how effective did you feel your MDT care was?

PLEASE CHECK ONE:

􀂅Very Effective

􀂅Effective

[Please proceed to Question 28]

􀂅neither effective nor ineffective

􀂅Ineffective

􀂅Very Ineffective

Question 27d

What were the reasons for your MDT care being ineffective?

PLEASE CHECK ALL THAT APPLY

􀂅 There was no referral to a local team in primary care, e.g. a local physiotherapist

􀂅 There was no referral to tertiary care (e.g. a specialist urologist)

􀂅 Referral to tertiary care (e.g. a specialist urologist) was not effective

􀂅 The MDT did not understand my needs

􀂅 There was no treatment even after being seen by the MDT

􀂅 Other [Please specify in the box below]

􀂅 Unsure

TEXT BOX

Please enter your response here

Questions 28–34

Please rate your agreement with the following 12 statements on a scale of 1-6:

1. Strongly agree

2. Agree

3. neither agree or disagree

4. Disagree

5. Strongly Disagree

6. N/A

PLEASE CHECK ONE PER LINE:

1 2 3 4 5 6

28. GP understood how to manage my ataxia.

• • • • • • •

29. GP understood the treatments available for my ataxia.

• • • • • • •

30. Neurologists in a general neurology clinic (not at a specialist ataxia centre) understood how to manage my ataxia.

• • • • • • •

31. Neurologists in a general neurology clinic (not at the specialist ataxia centre) understood the treatments available for my ataxia.

• • • • • • •

32 Neurologist at the specialist ataxia centre understood how to manage my ataxia.

• • • • • • •

33. Neurologists at the specialist ataxia centre understood the treatments available for my ataxia.

• • • • • • •

34. Healthcare workers in Accident &Emergency understood how my ataxia might affect the treatment provided to me.

Question 35

Please rate your agreement with the following statement:

The care I received during the time I spent in A&E could have been better, for example if they knew more about my condition, if patients had a card with them with key information about the condition to show to A&E staff.

PLEASE CHECK ONE

Strongly Agree

Agree Neither

agree

Nor disagree

Disagree

Strongly Disagree

N/A

Question 36

Have you ever been referred for treatment of any of the following symptoms as a result of your ataxia?

Please tick the box corresponding to your answer in the row opposite each symptom. For example, if you are unsure you would tick box D

A. Yes, I have experienced this symptom and have been referred for its treatment

B. No, I have experienced this symptom but have not been referred for treatment

C. No, I have never experienced this symptom

D. Unsure

PLEASE CHECK ONE PER LINE:

Symptom A B C D

Pain • • • •

Heart problems • • • • 

Gastroenterological problems • • • • 

Sexual dysfunction • • • • 

Swallowing • • • • 

Hearing problems • • • • 

Eye symptoms • • • • 

Sleep disturbances • • • • 

Speech problems • • • • 

Depression • • • • 

Other mental health issues • • • • 

Fatigue • • • • 

Other [Please specify in the box below] • • • • 

TEXT BOX

Please enter your response here

Question 37

Please tell us about the symptoms you have experienced in the LAST YEAR and the referral you have had for treatment of these symptoms:

PLEASE CHECK ALL THAT APPLY

Dystonia: uncontrolled and sometimes painful muscle movements causing unusual body positions

Stiffness/rigidity of your legs or muscles cramps

Spasms

Bladder problems

Dystonia

Tremors

I have experienced this symptom

I have been referred by the GP for treatment of this symptom

I have been referred by another primary care professional for treatment of this symptom

I have been referred by hospital neurologist (non-specialist) for treatment of this symptom

I have been referred by neurologist at Specialist ataxia centre for treatment of this symptom

I have received treatment for this symptom outside the specialist ataxia centre

I have received treatment for this symptom at the specialist ataxia centre

I have not sought treatment for this symptom

I have not been offered treatment for this symptom

Questions 38-43

If you have experienced the following symptoms in THE PAST YEAR and received treatment, how was the symptom treated:

PLEASE CHECK ALL THAT APPLY

38. Stiffness/rigidity of your legs or muscles

• Drug therapy (by mouth or injection)

• Physiotherapy

• Occupational therapy

• Alternative therapies (e.g. homeopathy, acupuncture, reflexology)

• Exercise (e.g. visiting the gym, pilates classes)

• Other (please specify in box below)

• Unsure

39. Cramps

• Drug therapy (by mouth or injection)

• Physiotherapy

• Occupational therapy

• Alternative therapies (e.g. homeopathy, acupuncture, reflexology)

• Exercise (e.g. visiting the gym, pilates classes)

• Other (please specify in box below)

• Unsure

40. Spasms

• Drug therapy (by mouth or injection)

• Physiotherapy

• Occupational therapy

• Surgery

• Alternative therapies (e.g. homeopathy, acupuncture, reflexology)

• Exercise (e.g. visiting the gym, pilates classes)

• Other (please specify in box below)

• Unsure

41. Bladder problems

• Drug therapy (by mouth or injection)

• Catheterisation

• Nerve stimulation

• Exercise (e.g. visiting the gym, pilates classes)

• Other (please specify in box below)

• Unsure

42. Dystonia

• Drug therapy (by mouth or injection)

• Occupational therapy

• Physiotherapy

• Exercise (e.g. visiting the gym, pilates classes)

• Other (please specify in box below)

• Unsure

43. Tremors

• Drug therapy (by mouth or injections)

• Occupational therapy

• Exercise (e.g. visiting the gym, pilates classes)

• Other (please specify in box below)

• Unsure

Question 44

If you have experienced the following symptoms in THE PAST YEAR and received treatment, how well do you feel that the symptom was managed?

PLEASE CHECK ONE PER LINE

Best it could be

Very well

adequately

poorly

Very poorly

N/A

Stiffness/rigidity

Cramps

spasms

Bladder problems

dystonia

tremors

Question 45

Overall, including all symptoms mentioned above and any other symptoms that you experience as a result of your ataxia, do you feel your symptoms are well managed?

Best it could be Very well Adequately poorly Very poorly N/A

Question 46

How well do you feel your care reflects your needs?

Best it could be Very well Adequately poorly Very poorly N/A

Question 47

How do you feel your care could be improved?

Please check all that apply

• More information about my condition

• More information about available treatments

•More help to make me feel in control of my condition (i.e. to cope better)

• Knowing my specific diagnosis earlier

• Better management of my symptoms

• Better practical advice on living with the condition

•Better access to therapies (e.g. physiotherapy, speech and language therapy, occupational therapy)

• More information on help adapting my home

• Help communicating with my employer

• More information on the genetics of my condition/whether my children or grandchildren would be at risk of inheriting ataxia

• Continuing the same level of care in my home if I cannot attend a SAC any longer

• I am satisfied with my care, it does not need improvement

• Other [Please specify in the box below]

• Unsure

TEXT BOX

Please enter your response here
