# Supplementary material for: Friedreich's ataxia patient pathway in Europe
Source: Front Health Serv. 2026 May 28;6:1817584. doi: 10.3389/frhs.2026.1817584 (PMC13254176; doi:10.3389/frhs.2026.1817584)
Supplement: Supplementary file 4 [file Supplementaryfile4.docx]

Supplementary Figure 1a: Time since the first diagnosis in the UK

Supplementary Figure 1b: Time since the first diagnosis in Germany

1-5 years

6-10 years

11-15 years

16-20 years

21-25 years

26-30 years

31-35 years

36-40 years

Supplementary Figure 1c: Time since the first diagnosis in Italy

36-40 years

31-35 years

26-30 years

21-25 years

16-20 years

11-15 years

6-10 years

1-5 years
