# Supplementary material for: Friedreich's ataxia patient pathway in Europe
Source: Front Health Serv. 2026 May 28;6:1817584. doi: 10.3389/frhs.2026.1817584 (PMC13254176; doi:10.3389/frhs.2026.1817584)
Supplement: Supplementary file 5 [file Table1.docx]

Supplementary Table 1a: Level of mobility – German cohort

| **Answer choices** | **Responses N (%)** |
| --- | --- |
| No functional restriction | 0 |
| Mild impairment, able to run, I can walk without restriction | 0 |
| Moderate impairment, unable to run, I can walk a limited distance without aids | 2 (14.3) |
| I need one stick to walk | 1 (7.1) |
| In the house I need two sticks / a rollator, I need a wheelchair outside | 2 (14.3) |
| I can’t walk and I need a wheelchair, still independent | 4 (28.6) |
| I can’t walk and I need a wheelchair, dependent | 5 (35.7) |
| I am bedridden | 0 |
| Total | 14 (100) |

Supplementary Table 1b: Level of mobility – Italian cohort

| **Answer choices** | **Responses N (%)** |
| --- | --- |
| No functional restriction | 0 |
| Mild impairment, able to run, I can walk without restriction | 0 |
| Moderate impairment, unable to run, I can walk a limited distance without aids | 7 (12.5) |
| I need one stick to walk | 2 (3.6) |
| In the house I need two sticks / a rollator, I need a wheelchair outside | 4 (7.1) |
| I can’t walk and I need a wheelchair, still independent | 7 (12.5) |
| I can’t walk and I need a wheelchair, dependent | 35 (62.5) |
| I am bedridden | 1 (1.8) |
| Total | 56 (100) |
