# Supplementary material for: Friedreich's ataxia patient pathway in Europe
Source: Front Health Serv. 2026 May 28;6:1817584. doi: 10.3389/frhs.2026.1817584 (PMC13254176; doi:10.3389/frhs.2026.1817584)
Supplement: Supplementary file 6 [file Table2.docx]

Supplementary Table 2: where the confirmation of FA diagnosis was made

2a – Germany

| **Answer choices** | **Responses N (%)** |
| --- | --- |
| In a specialist ataxia centre | 5 (35.7) |
| In a non-specialist service | 5 (35.7) |
| Other (please specify in the box below) | 3 (21.4) |
| Unsure | 1 (7.2) |
| Total | 14 (100) |

*Other: Human Genetics Göttingen; Laboratories in Bochum; Uni hospital Homburg (Saar)*

2b – Italy

| **Answer choices** | **Responses N (%)** |
| --- | --- |
| In a specialist ataxia centre | 33 (58.9) |
| In a non-specialist service | 20 (35.7) |
| Other (please specify in the box below) | 2 (3.6) |
| Unsure | 1 (1.8) |
| Total | 55 (100) |

*Other: Bari Hospital, University Hospital Siena*
