# Supplementary material for: Friedreich's ataxia patient pathway in Europe
Source: Front Health Serv. 2026 May 28;6:1817584. doi: 10.3389/frhs.2026.1817584 (PMC13254176; doi:10.3389/frhs.2026.1817584)
Supplement: Supplementary file 7 [file Table3.docx]

Supplementary Table 3: Primary contact for ataxia

1. UK

| **Attendance to SAC**  **n (%)** | **GP** | **Neurologist (not at SAC)** | **Neurologist at SAC** | **Other** | **Unsure** | **Total** |
| --- | --- | --- | --- | --- | --- | --- |
| **Yes currently** | 0 (0) | 0 (0) | 3 (100) | 0 (0) | 0 (0) | **3 (100)** |
| **No to SAC** | 3 (33.3) | 5 (66.7) | 0 (0) | 0 (0) | 0 (0) | **8 (100)** |
| **Used to SAC** | 1 (33.3) | 1 (33.3) | 1 (33.4) | 0 (0) | 0 (0) | **3 (100)** |
| **Unsure** | 0 (0) | 3 (60) | 1 (20) | 0 (0) | 1 (20) | **5 (100)** |
| **Total** | **4 (21)** | **9 (47.4)** | **5 (26.3)** | **0 (0)** | **1 (5.3)** | **19 (100)** |

1. Germany

| **Answer choices** | **Responses N (%)** |
| --- | --- |
| General practitioner/family doctor | 5 (35.7) |
| Neurologist not at a SAC | 3 (21.4) |
| Neurologist at a SAC | 6 (42.9) |
| Other | 0 |
| Unsure | 0 |
| Total | 14 (100) |

1. Italy

| **Answer choices** | **SAC group** | **Non SAC** | **Used to SAC** | **Responses N (%)** |
| --- | --- | --- | --- | --- |
| General practitioner/family doctor | 0 | 1 (33.3) | 1 (11.1) | 2 (4.9) |
| Neurologist not at a SAC | 2 (6.9) | 0 | 5 (55.6) | 7 (17) |
| Neurologist at a SAC | 27 (93.1) | 0 | 3 (33.4) | 30 (73.2) |
| Other | 0 | 2 (66.7) | 0 | 2 (4.9) |
| Unsure | 0 | 0 | 0 | 0 (0) |
| Total | 29 (100) | 3 (100) | 9 (100) | 41 (100) |

Other: neurologist-geneticist ataxia specialist, I am not really followed, AFFILIATED REHABILITATION CENTRE NEUROLOGIST
