# Supplementary material for: Friedreich's ataxia patient pathway in Europe
Source: Front Health Serv. 2026 May 28;6:1817584. doi: 10.3389/frhs.2026.1817584 (PMC13254176; doi:10.3389/frhs.2026.1817584)
Supplement: Supplementary file 8 [file Table4.docx]

Supplementary Table 4: Reasons why people stopped going to a SAC

| **Answer choices N (%)** | **UK** | **Germany** | **Italy** |
| --- | --- | --- | --- |
| Problems with travelling/transport | 2 (66.7) | 0 (0) | 2 (20) |
| Did not find it useful | 1 (33.3) | 0 (0) | 2 (20) |
| Not referred again | 0 (0) | 0 (0) | 0 (0) |
| Equal care locally | 0 (0) | 0 (0) | 3 (30) |
| Other reasons | 0 (0) | 1 (100) | 1 (10) |
| Unsure | 0 (0) | 0 (0) | 2 (20) |
| Used to receive care at the Newcastle specialist centre, but the centre is no longer available and I have not been referred to another | 0 (0) | _ | _ |
| Total | 3 (100) | 1 (100) | 10 (100) |

Other reason in Germany: appointment cancelled due to Covid; other reason in Italy: transfer to another service
