# Supplementary material for: Friedreich's ataxia patient pathway in Europe
Source: Front Health Serv. 2026 May 28;6:1817584. doi: 10.3389/frhs.2026.1817584 (PMC13254176; doi:10.3389/frhs.2026.1817584)
Supplement: Supplementary file 9 [file Table5.docx]

Supplementary Table 5: Reasons why people never went to a SAC

| **Answer choices N (%)** | **UK** | **Italy** |
| --- | --- | --- |
| Current level of care is sufficient | 3 (25) | 0 (0) |
| I asked to be referred to a specialist ataxia centre but was refused by my doctor | 1 (8.3) | 0 (0) |
| The specialist ataxia centres are too far away for me to travel to | 4 (33.3) | 0 (0) |
| Did not wish to be referred | 1 (8.3) | 2 (40) |
| Other reasons | 2 (16.7) | 2 (40) |
| Unsure | 1 (8.3) | 1 (20) |
| Total | 12 (100) | 10 (100) |

Other reason in the UK: I went to John Radcliffe Hospital Ataxia Clinic, It’s never been suggested I attend an ataxia specialist centre

Other reason in Italy: a referral was not offered
