# Supplementary material for: Friedreich's ataxia patient pathway in Europe
Source: Front Health Serv. 2026 May 28;6:1817584. doi: 10.3389/frhs.2026.1817584 (PMC13254176; doi:10.3389/frhs.2026.1817584)
Supplement: Supplementary file 10 [file Table6.docx]

Supplementary Table 6: attendance at an MDT clinic

1. UK

| **Attendance to SAC**  **N (%)** | **YES** | **NO** | **Unsure** | **Total** |
| --- | --- | --- | --- | --- |
| Yes currently | 2 (66.7) | 1 (33.3) | 0 (0) | 3 (100) |
| No to SAC | 5 (50) | 5 (50) | 0 (0) | 10 (100) |
| Used to go to SAC | 3 (100) | 0 (0) | 0 (0) | 3 (100) |
| unsure | 1 (33.3) | 2 (66.7) | 0 (0) | 3 (100) |
| total | 11 (57.9) | 8 (42.1) | 0 (0) | 19 (100) |

1. Germany

| **Attendance to SAC**  **N (%)** | **YES** | **NO** | **Unsure** | **Total** |
| --- | --- | --- | --- | --- |
| Yes currently | 4 (40) | 5 (50) | 1 (10) | 10 (100) |
| Used to go to SAC | 0 (0) | 2 (100) | 0 (0) | 2 (100) |
| unsure | 0 (0) | 0 (0) | 0 (0) | 0 (0) |
| total | 4 (33.3) | 7 (58.3) | 1 (8.4) | 12 (100) |

1. Italy

| **Attendance to SAC**  **N (%)** | **YES** | **NO** | **Unsure** | **Total** |
| --- | --- | --- | --- | --- |
| Yes currently | 15 (57.7) | 8 (30.8) | 3 (11.5) | 26 (100) |
| Used to go to SAC | 5 (55.6) | 3 (33.3) | 1 (11.1) | 9 (100) |
| No to SAC | 1 (20) | 3 (60) | 1 (20) | 5 (100) |
| Unsure | 0 (0) | 0 (0) | 0 (0) | 0 (0) |
| total | 21 (52.5) | 14 (35) | 5 (12.5) | 40 (100) |
