# Supplementary material for: Friedreich's ataxia patient pathway in Europe
Source: Front Health Serv. 2026 May 28;6:1817584. doi: 10.3389/frhs.2026.1817584 (PMC13254176; doi:10.3389/frhs.2026.1817584)

Supplementary Table 7: Participants feedback on the visit to MDT

a-UK

|  | positive | negative | % positive | % negative |
| --- | --- | --- | --- | --- |
| YES to SAC | 2 | 0 | 100 | 0 |
| No to SAC | 3 | 2 | 60 * | 40 * |
| Used to SAC | 3 | 0 | 100 | 0 |
| total | 8 | 2 | 80 | 20 |
| total responses | | 10 |  |  |

* P<0.001

b- Germany

|  | positive | neutral | negative | % positive | %neutral | % negative |
| --- | --- | --- | --- | --- | --- | --- |
| YES to SAC | 3 | 1 | 0 | 75 | 25 | 0 |
| total responses | |  | 4 |  |  |  |

c- Italy


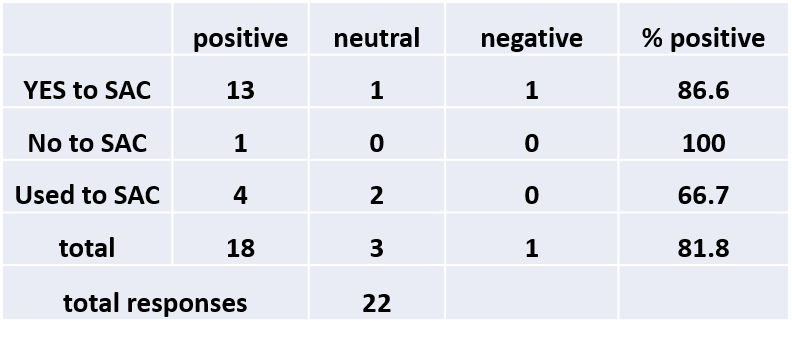

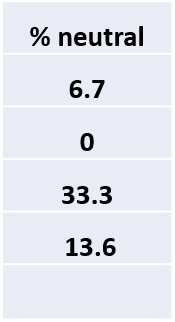

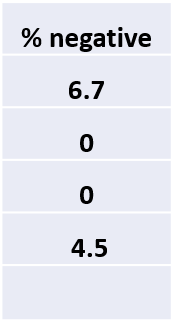

Supplement: Supplementary file 11 [file Table7.docx]
