# Supplementary material for: Friedreich's ataxia patient pathway in Europe
Source: Front Health Serv. 2026 May 28;6:1817584. doi: 10.3389/frhs.2026.1817584 (PMC13254176; doi:10.3389/frhs.2026.1817584)
Supplement: Supplementary file 12 [file Table8.docx]

Supplementary Tables 8: Comparison of SAC with non-SAC services in Germany and Italy for the following statements

1. Good understanding of the condition - Germany

|  | positive | neutral | negative | % positive | | % neutral | | % negative | |
| --- | --- | --- | --- | --- | --- | --- | --- | --- | --- |
| YES to SAC | 9 | 2 | 0 | 81.8 | | 18.2 | | 0 | |
| Used to SAC | 2 | 0 | 0 | 100 | | 0 | | 0 | |
|  |  |  |  |  | |  | |  | |
| total | 11 | 2 | 0 | 84.6 | | 15.4 | | 0 | |
| total responses | | 13 |  |  |  | |  | |  |

1. Good understanding of the condition - Italy

|  | **positive** | **neutral** | **negative** | **% positive** | **% neutral** | | **% negative** |
| --- | --- | --- | --- | --- | --- | --- | --- |
| YES to SAC | 25 | 3 | 0 | 89.3 | 10.7 | | 0 |
| Used to SAC | 7 | 2 | 0 | 77.8 | 22.2 | | 0 |
| total | 32 | 5 | 0 | 86.5 | 13.5 | | 0 |
| total responses | | 37 |  |  |  |  |  |

1. Makes you able to cope better with your condition - Germany

|  | positive | neutral | negative | % positive | | % neutral | | % negative | |
| --- | --- | --- | --- | --- | --- | --- | --- | --- | --- |
| YES to SAC | 6 | 5 | 0 | 54.5 | | 45.5 | | 0 | |
| Used to SAC | 1 | 1 | 0 | 50 | | 50 | | 0 | |
|  |  |  |  |  | |  | |  | |
| total | 7 | 6 | 0 | 53.8 | | 46.2 | | 0 | |
| total responses | | 13 |  |  |  | |  | |  |

1. Makes you able to cope better with your condition – Italy

|  | **positive** | **neutral** | **negative** | **% positive** | **% neutral** | | **% negative** |
| --- | --- | --- | --- | --- | --- | --- | --- |
| YES to SAC | 20 | 8 | 0 | 71.4 | 28.6 | | 0 |
| Used to SAC | 5 | 3 | 1 | 55.6 | 33.3 | | 11.1 |
|  |  |  |  |  |  | |  |
| total | 25 | 11 | 1 | 67.6 | 29.7 | | 2.7 |
| total responses | | 37 |  |  |  |  |  |

1. Gives practical advice on how to live better with my condition - Germany

|  | positive | neutral | negative | % positive | | % neutral | | % negative | |
| --- | --- | --- | --- | --- | --- | --- | --- | --- | --- |
| YES to SAC | 8 | 3 | 0 | 72.7 | | 27.3 | | 0 | |
| Used to SAC | 1 | 1 | 0 | 50 | | 50 | | 0 | |
|  |  |  |  |  | |  | |  | |
| total | 9 | 4 | 0 | 69.2 | | 30.8 | | 0 | |
| total responses | | 13 |  |  |  | |  | |  |

1. Gives practical advice on how to live better with my condition – Italy

|  | **positive** | **neutral** | **negative** | **% positive** | **% neutral** | | **% negative** | |
| --- | --- | --- | --- | --- | --- | --- | --- | --- |
| YES to SAC | 21 | 7 | 0 | 75 | 25 | | 0 | |
| Used to SAC | 5 | 3 | 1 | 55.6 | 33.3 | | 11.1 | |
|  |  |  |  |  |  | |  | |
| total | 26 | 10 | 1 | 70.3 | 27 | | 2.7 | |
| total responses | | 37 |  |  |  |  | |  |

1. Provides medical advice on the management of your symptoms - Germany

|  | positive | neutral | negative | % positive | | % neutral | | % negative | |
| --- | --- | --- | --- | --- | --- | --- | --- | --- | --- |
| YES to SAC | 6 | 5 | 0 | 54.5 | | 45.5 | | 0 | |
| Used to SAC | 0 | 2 | 0 | 0 | | 100 | | 0 | |
|  |  |  |  |  | |  | |  | |
| total | 6 | 7 | 0 | 46.2 | | 53.8 | | 0 | |
| total responses | | 13 |  |  |  | |  | |  |

1. Provides medical advice on the management of your symptoms - Italy

|  | **positive** | **neutral** | **negative** | **% positive** | **% neutral** | | **% negative** |
| --- | --- | --- | --- | --- | --- | --- | --- |
| YES to SAC | 21 | 7 | 0 | 75 | 25 | | 0 |
| Used to SAC | 5 | 3 | 1 | 55.6 | 33.3 | | 11.1 |
|  |  |  |  |  |  | |  |
| total | 26 | 10 | 1 | 70.3 | 27 | | 2.7 |
| total responses | | 37 |  |  |  |  |  |
