# Supplementary material for: Friedreich's ataxia patient pathway in Europe
Source: Front Health Serv. 2026 May 28;6:1817584. doi: 10.3389/frhs.2026.1817584 (PMC13254176; doi:10.3389/frhs.2026.1817584)
Supplement: Supplementary file 13 [file Table9.docx]

Supplementary Table 9a-r: Participants’ feedback on healthcare services visits

a - Primary care HCPs understood how to manage my ataxia - UK

|  | positive | negative | % positive | % negative |
| --- | --- | --- | --- | --- |
| YES to SAC | 1 | 2 | 33.3 | 66.7 |
| No to SAC | 4 | 6 | 40 | 60 |
| Used to SAC | 3 | 0 | 100 | 0 |
| total | 8 | 8 | 50 | 50 |
| total responses | | **16** |  |  |

b- Primary care HCPs understood how to manage my ataxia - Germany

|  | positive | neutral | negative | % positive | % neutral | % negative |
| --- | --- | --- | --- | --- | --- | --- |
| YES to SAC | 6 | 3 | 1 | 60 | 30 | 10 |
| Used to SAC | 1 | 1 | 0 | 50 | 50 | 0 |
| total | 7 | 4 | 1 | 58.3 | 33.3 | 8.4 |
| total responses | |  | **12** |  |  |  |

c- Primary care HCPs understood how to manage my ataxia - Italy

|  | positive | neutral | negative | % positive | % neutral | % negative |
| --- | --- | --- | --- | --- | --- | --- |
| YES to SAC | 12 | 7 | 5 | 50 | 29.2 | 20.8 |
| No to SAC | 3 | 0 | 2 | 60 | 0 | 40 |
| Used to SAC | 0 | 3 | 4 | 0 | 42.9 | 57.1 |
| total | 15 | 10 | 11 | 41.7 | 27.8 | 30.5 |
| total responses | |  | 36 |  |  |  |

d- Primary care HCPs understood the treatments available for my ataxia UK

|  | positive | negative | % positive | % negative |
| --- | --- | --- | --- | --- |
| YES to SAC | 2 | 1 | 66.7 | 33.3 |
| No to SAC | 3 | 7 | 30.0 | 70.0 |
| Used to SAC | 2 | 1 | 66.7 | 33.3 |
| total | 8 | 9 |  |  |
| total responses | | **17** |  |  |

e- Primary care HCPs understood the symptoms of my ataxia - Germany

|  | positive | neutral | negative | % positive | % neutral | % negative |
| --- | --- | --- | --- | --- | --- | --- |
| YES to SAC | 6 | 3 | 1 | 60 | 30 | 10 |
| Used to SAC | 1 | 1 | 0 | 50 | 50 | 0 |
| total | 7 | 4 | 1 | 58.3 | 33.3 | 8.4 |
| total responses | |  | **12** |  |  |  |

f- Primary care HCPs understood the symptoms of my ataxia - Italy

|  | positive | neutral | negative | % positive | % neutral | % negative |
| --- | --- | --- | --- | --- | --- | --- |
| YES to SAC | 12 | 6 | 8 | 46.2 | 25 | 30.8 |
| No to SAC | 3 | 0 | 2 | 60 | 0 | 40 |
| Used to SAC | 2 | 3 | 3 | 25 | 37.5 | 37.5 |
| total | 17 | 9 | 13 | 43.6 | 23.1 | 33.3 |
| total responses | |  | 39 |  |  |  |

g- Secondary care HCPs understood how to manage my ataxia - UK

|  | positive | negative | % positive | % negative |
| --- | --- | --- | --- | --- |
| YES to SAC | 1 | 2 | 33.3 | 66.7 |
| No to SAC | 6 | 4 | 60 | 40 |
| Used to SAC | 3 | 0 | 100 | 0 |
| total | 10 | 6 | 62.5 | 37.5 |
| total responses | | **16** |  |  |

h- Secondary care HCPs understood how to manage my ataxia - Germany

|  | positive | neutral | negative | % positive | % neutral | % negative |
| --- | --- | --- | --- | --- | --- | --- |
| YES to SAC | 6 | 0 | 1 | 85.7 | 0 | 14.3 |
| Used to SAC | 2 | 0 | 0 | 100 | 0 | 0 |
| total | 8 | 0 | 1 | 88.9 | 0 | 11.1 |
| total responses | |  | **9** |  |  |  |

i- Secondary care HCPs understood how to manage my ataxia - Germany

|  | positive | neutral | negative | % positive | % neutral | % negative |
| --- | --- | --- | --- | --- | --- | --- |
| YES to SAC | 16 | 6 | 2 | 66.7 | 25 | 8.3 |
| No to SAC | 2 | 3 | 0 | 40 | 60 | 0 |
| Used to SAC | 2 | 4 | 1 | 28.6 | 57.1 | 14.3 |
| total | 20 | 13 | 3 | 55.6 | 36.1 | 8.3 |
| total responses | |  | 36 |  |  |  |

j- Secondary care HCPs understood the treatments available for my ataxia - UK

|  | positive | negative | % positive | % negative |
| --- | --- | --- | --- | --- |
| YES to SAC | 1 | 2 | 33.3 | 66.7 |
| No to SAC | 5 | 5 | 50 | 50 |
| Used to SAC | 3 | 0 | 100 | 0 |
| total | 9 | 7 | 56.3 | 43.8 |
| total responses | | **16** |  |  |

k- Secondary care HCPs understood the treatments available for my ataxia - Germany

|  | positive | neutral | negative | % positive | % neutral | % negative |
| --- | --- | --- | --- | --- | --- | --- |
| YES to SAC | 6 | 0 | 1 | 85.7 | 0 | 14.3 |
| Used to SAC | 2 | 0 | 0 | 100 | 0 | 0 |
| total | 8 | 0 | 1 | 88.9 | 0 | 11.1 |
| total responses | |  | **9** |  |  |  |

l-Secondary care HCPs understood the treatments available for my ataxia - Italy

|  | positive | neutral | negative | % positive | % neutral | % negative |
| --- | --- | --- | --- | --- | --- | --- |
| YES to SAC | 15 | 5 | 4 | 62.5 | 20.8 | 16.7 |
| No to SAC | 2 | 3 | 0 | 40 | 60 | 0 |
| Used to SAC | 1 | 4 | 1 | 16.7 | 66.6 | 16.7 |
| total | 18 | 12 | 5 | 51.4 | 34.3 | 14.3 |
| total responses | |  | 35 |  |  |  |

m- Specialists at SAC understood how to manage my ataxia - UK

|  | positive | negative | % positive | % negative |
| --- | --- | --- | --- | --- |
| YES to SAC | 3 | 0 | 100 | 0 |
| Used to SAC | 2 | 0 | 100 | 0 |
| total | 5 | 0 | 100 | 0 |
| total responses | | **5** |  |  |

n- Specialists at SAC understood how to manage my ataxia - Germany

|  | positive | neutral | negative | % positive | % neutral | % negative |
| --- | --- | --- | --- | --- | --- | --- |
| YES to SAC | 10 | 0 | 0 | 100 | 0 | 0 |
| Used to SAC | 2 | 0 | 0 | 100 | 0 | 0 |
| total | 12 | 0 | 0 | 100 | 0 | 0 |
| total responses | |  | **12** |  |  |  |

o- Specialists at SAC understood how to manage my ataxia - Italy

|  | positive | neutral | negative | % positive | % neutral | % negative |
| --- | --- | --- | --- | --- | --- | --- |
| YES to SAC | 20 | 5 | 1 | 76.9 | 19.2 | 3.9 |
| Used to SAC | 6 | 2 | 1 | 66.7 | 22.2 | 11.1 |
| total | 26 | 7 | 2 | 72.5 | 22.5 | 5 |
| total responses | |  | 35 |  |  |  |

p- Specialists at SAC understood the treatment available for my ataxia - UK

|  | positive | negative | % positive | % negative |
| --- | --- | --- | --- | --- |
| YES to SAC | 3 | 0 | 100 | 0 |
| Used to SAC | 1 | 0 | 100 | 0 |
| total | 4 | 0 | 100 | 100 |
| total responses | | **4** |  |  |

q-Specialists at SAC understood the treatment available for my ataxia - Germany

|  | positive | neutral | negative | % positive | % neutral | % negative |
| --- | --- | --- | --- | --- | --- | --- |
| YES to SAC | 10 | 0 | 0 | 100 | 0 | 0 |
| Used to SAC | 2 | 0 | 0 | 100 | 0 | 0 |
| total | 12 | 0 | 0 | 100 | 0 | 0 |
| total responses | |  | **12** |  |  |  |

r-Specialists at SAC understood the treatment available for my ataxia - Italy

|  | positive | neutral | negative | % positive | % neutral | % negative |
| --- | --- | --- | --- | --- | --- | --- |
| YES to SAC | 20 | 5 | 1 | 76.9 | 19.2 | 3.9 |
| Used to SAC | 6 | 2 | 1 | 66.7 | 22.2 | 11.1 |
| total | 26 | 7 | 2 | 72.5 | 20 | 7.5 |
| total responses | |  | 35 |  |  |  |
