# Supplementary material for: Friedreich's ataxia patient pathway in Europe
Source: Front Health Serv. 2026 May 28;6:1817584. doi: 10.3389/frhs.2026.1817584 (PMC13254176; doi:10.3389/frhs.2026.1817584)
Supplement: Supplementary file 14 [file Table10.docx]

Supplementary Table 10: Feedback about the overall symptoms management of the participants

a-In the UK


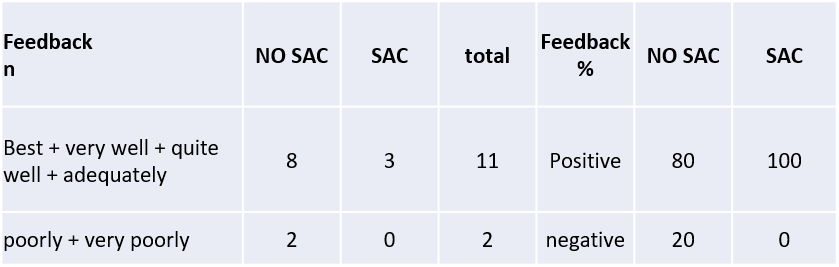


b- in Germany


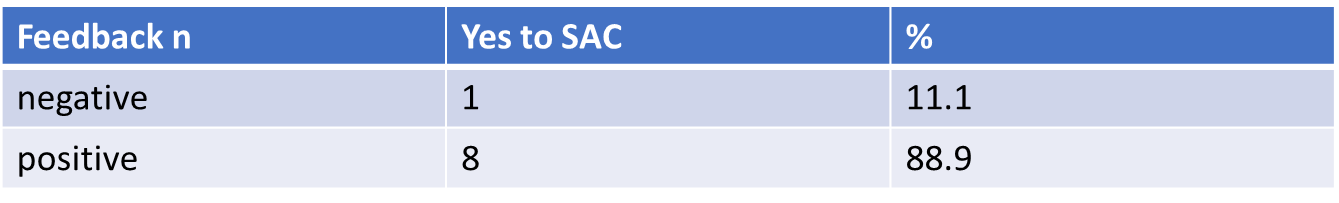


c- in Italy

| **Feedback**  **n** | **NO SAC** | **SAC** | **total** | **Feedback %** | **NO SAC** | **SAC** |
| --- | --- | --- | --- | --- | --- | --- |
| Best + very well + adequately | 2 | 15 | 17 | Positive | 100 | 68 |
| poorly + very poorly | 0 | 7 | 7 | negative | 0 | 32 |
