# Supplementary material for: Friedreich's ataxia patient pathway in Europe
Source: Front Health Serv. 2026 May 28;6:1817584. doi: 10.3389/frhs.2026.1817584 (PMC13254176; doi:10.3389/frhs.2026.1817584)
Supplement: Supplementary file 15 [file Table11.docx]

Supplementary Table 11: Feedback about the overall level of care received by participants being adapted to their needs

1. In the UK

| **Feedback**  **n** | **NO SAC** | **SAC** | **total** | **Feedback %** | **NO SAC** | **SAC** |
| --- | --- | --- | --- | --- | --- | --- |
| Best + very well + quite well + adequately | 7 | 3 | 10 | Positive | 70 | 100 |
| poorly + very poorly | 3 | 0 | 3 | negative | 30 | 0 |

1. In Germany


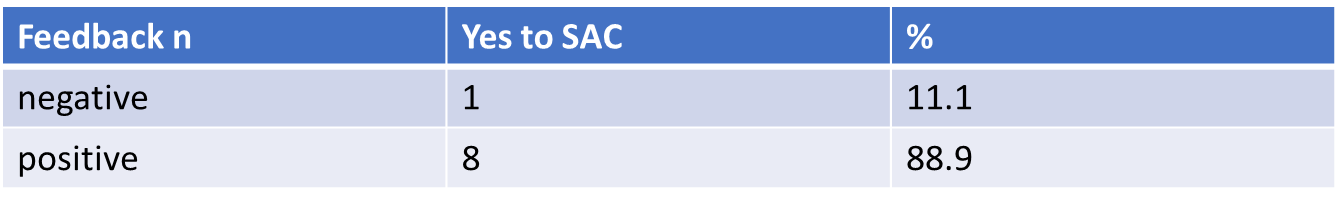


1. In Italy

| **Feedback**  **n** | **NO SAC** | **SAC** | **total** | **Feedback %** | **NO SAC** | **SAC** |
| --- | --- | --- | --- | --- | --- | --- |
| Best + very well + adequately | 1 | 13 | 14 | Positive | 33 | 59 |
| poorly + very poorly | 2 | 9 | 11 | negative | 67 | 41 |
