# Supplementary material for: Friedreich's ataxia patient pathway in Europe
Source: Front Health Serv. 2026 May 28;6:1817584. doi: 10.3389/frhs.2026.1817584 (PMC13254176; doi:10.3389/frhs.2026.1817584)
Supplement: Supplementary file 16 [file Table12.docx]

Supplementary Table 12: Feedback on how to improve the care

| **Answer choices** | **Responses UK N (%)** | **Responses Germany N (%)** | **Responses Italy N (%)** |
| --- | --- | --- | --- |
| More information about my condition | 8 (10.5) | 2 (7.7) | 14 (11.5) |
| More information about available treatments | **13 (17.1)** | - | **24 (19.7)** |
| More help so I can feel in control of my disease (to cope better) | 11 (14.5) | 1 (3.85) | 9 (7.4) |
| Knowing my specific diagnosis earlier | 3 (4 | 2 (7.7) | 5 (4.1) |
| Better management of my symptoms | 5 (6.6) | **3 (11.5)** | 10 (8.2) |
| Better practical advice on living with my condition | **9 (11.8)** | **6 (23.1)** | **17 (13.9)** |
| Better access to therapies (physiotherapy, speech therapy, occupational therapy) | **10 (13.2)** | **3 (11.5)** | **19 (15.6)** |
| More information on help adapting my home | 8 (10.5) | **3 (11.5)** | 6 (4.9) |
| Help in communicating with my employer | 1 (1.3) | 0 (0) | 1 (0.8) |
| More information about the genetics of my condition/ whether my children or grandchildren are at risk of inheriting ataxia | 2 (2.6) | 2 (7.7) | 2 (1.6) |
| Continuing the same level of care in my home if I am not longer able to visit an ataxia specialist centre | 6 (7.9) | 1 (3.85) | 13 (10.7) |
| I am satisfied with my care and do not need improvement | 0 (0) | 2 (7.7) | 1 (0.8) |
| Other (comment: repetitive conflicts with health insurance about the cover of aid costs) | - | 1 (3.85) | 1 (0.8) |
| Unsure | 0 (0) | 0 (0) | 0 (0) |
| I do not know | 0 (0) | - | 0 (0) |
| Total number of respondents | 76 (100) | 26 (100) | 122 (100) |
