# Supplementary material for: Friedreich's ataxia patient pathway in Europe
Source: Front Health Serv. 2026 May 28;6:1817584. doi: 10.3389/frhs.2026.1817584 (PMC13254176; doi:10.3389/frhs.2026.1817584)
Supplement: Supplementary file 17 [file Table13.docx]

Supplementary Table 13. Mode of transport and time taken to travel to the neurologist based in the general neurology clinic

| **Travel time to visit the neurologist based in the general neurology clinic (one way)** | | | | |
| --- | --- | --- | --- | --- |
| **UK** | **Patients who reported never attending a SAC (N=11)** | | **Patients who reported attending a SAC currently (N=3)** | |
|  | **N** | **%** | **N** | **%** |
| Less than 1 hour | 9 | 82 | 2 | 100 |
| 1 to 2 hours | 2 | 18 |  |  |
| 2 to 3 hours |  |  |  |  |
| 3 to 4 hours |  |  |  |  |
| More than 4 hours |  |  |  |  |
| Not applicable |  |  |  |  |
| Unsure |  |  |  |  |
| **ITALY** | **Patients who reported never attending a SAC (N=5)** | | **Patients who reported attending a SAC currently (N=26)** | |
|  | **N** | **%** | **N** | **%** |
| Less than 1 hour | 2 | 40 | 15 | 58 |
| 1 to 2 hours |  |  | 8 | 31 |
| 2 to 3 hours | 1 | 20 | 1 | 4 |
| 3 to 4 hours |  |  |  |  |
| More than 4 hours | 1 | 4 |  |  |
| Not applicable | 1 | 20 | 2 | 8 |
| Unsure | 1 | 20 |  |  |
| **GERMANY** | **Patients who reported never attending a SAC (N=0)** | | **Patients who reported attending a SAC currently (N=13)** | |
| Less than 1 hour |  |  | 8 | 62 |
| 1 to 2 hours |  |  | 1 | 8 |
| 2 to 3 hours |  |  | 1 | 8 |
| 3 to 4 hours |  |  |  |  |
| More than 4 hours |  |  |  |  |
| Not applicable |  |  | 3 | 23 |
| Unsure |  |  |  |  |
| **Mode of transport mainly used to visit the neurologist based in the general neurology clinic** | | | | |
| **ITALY** | **Patients who reported never attending a SAC (N=5)** | | **Patients who reported attending a SAC currently (N=25)** | |
|  | **N** | **%** | **N** | **%** |
| Bus |  |  |  |  |
| Car | 4 | 80 | 24 | 96 |
| Taxi | 1 | 20 |  |  |
| Train |  |  |  |  |
| Other |  |  |  |  |
| Unsure |  |  | 1 | 4 |

SAC, specialist ataxia centre; N, number of participants who responded to that question.
